# Involvement of a Membrane-Bound Amphiphilic Helix in Substrate Discrimination and Binding by an *Escherichia coli* S2P Peptidase RseP

**DOI:** 10.3389/fmicb.2020.607381

**Published:** 2020-11-27

**Authors:** Takuya Miyake, Yohei Hizukuri, Yoshinori Akiyama

**Affiliations:** Institute for Frontier Life and Medical Sciences, Kyoto University, Kyoto, Japan

**Keywords:** metallopeptidase, PDZ domain, regulated intramembrane proteolysis, extracytoplasmic stress response, site-2 protease, substrate recognition and specificity, amphipathic helix, exosite

## Abstract

Intramembrane proteases (IMPs) are a unique class of proteases that catalyze the proteolysis within the membrane and regulate diverse cellular processes in various organisms. RseP, an *Escherichia coli* site-2 protease (S2P) family IMP, is involved in the regulation of an extracytoplasmic stress response through the cleavage of membrane-spanning anti-stress-response transcription factor (anti-σ^E^) protein RseA. Extracytoplasmic stresses trigger a sequential cleavage of RseA, in which first DegS cleaves off its periplasmic domain, and RseP catalyzes the second cleavage of RseA. The two tandem-arranged periplasmic PDZ (PDZ tandem) domains of RseP serve as a size-exclusion filter which prevents the access of an intact RseA into the active site of RseP IMP domain. However, RseP’s substrate recognition mechanism is not fully understood. Here, we found that a periplasmic region of RseP, located downstream of the PDZ tandem, contains a segment (named H1) predicted to form an amphiphilic helix. Bacterial S2P homologs with various numbers of PDZ domains have a similar amphiphilic helix in the corresponding region. We demonstrated that the H1 segment forms a partially membrane-embedded amphiphilic helix on the periplasmic surface of the membrane. Systematic and random mutagenesis analyses revealed that the H1 helix is important for the stability and proteolytic function of RseP and that mutations in the H1 segment can affect the PDZ-mediated substrate discrimination. Cross-linking experiments suggested that H1 directly interacts with the DegS-cleaved form of RseA. We propose that H1 acts as an adaptor required for proper arrangement of the PDZ tandem domain to perform its filter function and for substrate positioning for its efficient cleavage.

## Introduction

Intramembrane proteases (IMPs) are a unique class of proteases that exhibit catalytic residues for peptide-bond hydrolysis within the lipid bilayer. The four families of IMPs include site-2 protease (S2P; zinc metallopeptidase), rhomboid (serine protease), presenilin/signal peptide peptidase (SPP; aspartyl protease), and Rce1 (glutamyl protease; Sun et al., 2016; Beard et al., 2019). The first three families of IMPs cleave a transmembrane (TM) segment of membrane proteins (Wolfe, 2009; Strisovsky, 2016), while Rce1, the only member of the glutamyl IMPs, cleaves a soluble domain of the substrate. IMPs are involved in various cellular events in a wide range of organisms and often mediate transmembrane signaling by regulating the cleavage of a target protein in response to environmental changes (Brown et al., 2000; Kühnle et al., 2019).

The S2P family is well conserved among all kingdoms of life, from prokaryotes to higher eukaryotes (Chen and Zhang, 2010; Kroos and Akiyama, 2013; Rawson, 2013; Schneider and Glickman, 2013). Eukaryotic S2Ps, including human S2P, which was the first discovered member of this family, control cholesterol and fatty acid biosynthesis and endoplasmic reticulum (ER) stress responses through cleavage of membrane-bound transcription factors. While bacterial S2Ps play similar roles in the regulation of membrane protein/lipid biogenesis and stress responses, they also influence other cellular processes such as sporulation, pheromone production, and virulence.

*Escherichia coli* RseP, one of the most researched S2P family, was first shown to act as a regulator of an extracytoplasmic stress response *via* the cleavage of the anti-stress-response transcription factor (anti-σ^E^) protein RseA (Ades, 2008; Hizukuri et al., 2013). Under low-stress growth conditions, σ^E^ is inactivated by interacting with the cytoplasmic domain of RseA, a type II (N_IN_-C_OUT_) single-spanning membrane protein. Heat or other environmental stressors induce the activation of a membrane-anchored protease DegS and release from RseA of RseB, a negative regulator that binds to the RseA periplasmic domain, leading to the DegS-catalyzed first (site-1) cleavage of RseA in its periplasmic domain. This first cleavage triggers the RseP-induced second (site-2) cleavage within the RseA TM segment (Akiyama et al., 2004; Lima et al., 2013). The site-2 cleavage causes the liberation of the RseA cytoplasmic fragment complexed with σ^E^ and the final activation of σ^E^ as a result of the degradation of the RseA cytoplasmic domain by cytoplasmic proteases such as ClpXP. Ultimately, the activation of σ^E^ induces transcription of stress genes. RseP was also shown to contribute to the quality control of the cytoplasmic membrane by eliminating remnant signal peptides that are generated during the translocation of secretory proteins (Saito et al., 2011).

The S2P family is suggested to have a characteristic core domain composed of three transmembrane-spanning helical (TMH1–TMH3) segments. TMH1 and TMH3 segments contain a zinc binding motif HExxH and a zinc ligand Asp residue, respectively. This family of proteases can be classified into several subgroups: The members of Group I possess one or more PDZ (PSD-95/Dlg/ZO-1) domain(s), which is generally involved in protein-protein interactions in many prokaryotic and eukaryotic proteins, between TMH2 and TMH3, while the homologs from Group II to IV do not (Kinch et al., 2006; Kroos and Akiyama, 2013). *Escherichia coli* RseP is part of the Group I S2P protease with four TM segments and two PDZ domains (PDZ-N and PDZ-C; Inaba et al., 2008; Li et al., 2009). TM1–TM3 of RseP corresponds to TMH1–TMH3 of the core domain (Feng et al., 2007). The two PDZ tandem domains of RseP are located in the periplasmic region between TM2 and TM3 (Inaba et al., 2008; Hizukuri et al., 2014). The PDZ tandem of RseP was first shown to be involved in negative regulation of substrate cleavage (Kanehara et al., 2003; Inaba et al., 2008; Hizukuri and Akiyama, 2012) through mutagenic analyses, demonstrating that impaired PDZ tandem caused unregulated cleavage of full-length RseA by RseP. A subsequent study showed that the PDZ tandem would act as a size-exclusion filter to prevent the access of intact (full-length) RseA to the recessed active site in the membrane-embedded protease domain (Hizukuri et al., 2014). The DegS-cleaved form of RseA, which has lost most of its periplasmic domain, can pass through the PDZ filter and gain access to the intramembrane active site of RseP. It has been suggested that the single PDZ domain of *Bacillus subtilis* S2P homolog, RasP, might also act as a size-exclusion filter (Parrell et al., 2017).

In addition to the PDZ tandem, three other RseP structural elements involved in the process of the substrate recognition and cleavage have been identified. The membrane-reentrant β-loop (MRE β-loop), which is highly conserved among the members of S2P Groups I and III, including RseP (Group I), *B. subtilis* SpoIVFB (Group III), and *Methanocaldococcus jannaschii* S2P (Group III), has been proposed to form a membrane-embedded β-hairpin-like-structure (Zhang et al., 2013; Akiyama et al., 2015). The MRE β-loop directly binds to a substrate and induces its conformational change to an extended form for presentation to the active site (Akiyama et al., 2015). The N-terminal part of the first cytoplasmic loop (C1N), located adjacent to the MRE β-loop, contains a region with a conserved GFG motif (Akiyama et al., 2017) that directly binds to a substrate and supports the function of the downstream MRE β-loop in substrate recognition by RseP. In addition, the short loop region of RseP TM3 has also been shown to interact with RseA (Koide et al., 2008). A study using SpoIVFB suggested that this short loop region forms a hydrophobic face of the active site pocket that may assist the interaction of the MRE β-loop with an extended substrate at the opposite side (Halder et al., 2017). While multiple binding sites for a substrate have been identified for RseP, it remains unknown how RseP specifically recognizes and delivers a substrate to the active site and how other parts of RseP are involved in these processes.

In this study, we focused on a periplasmic region (named the PDZ carboxyl terminal (PCT) region) located downstream of the PDZ domain of RseP. The corresponding regions of bacterial Group I S2Ps commonly contain a characteristic segment (named H1) that is predicted to form an amphiphilic helix. We showed that H1 forms a peripheral amphiphilic membrane helix that directly interacts with the DegS-cleaved form of RseA. Mutational study suggested that H1 contributes to the PDZ-mediated substrate discrimination. We propose that the H1 segment acts as an adaptor that mediates both the structural and functional interaction between the PDZ domains and the protease domain of RseP and supports the proper substrate positioning for cleavage.

## Materials and Methods

### Media

L medium (10 g/L Bacto Tryptone, 5 g/L yeast extract and 5 g/L NaCl; pH adjusted to 7.2 by using NaOH) and M9 medium (without CaCl_2_; Miller, 1972) supplemented with 2 μg/ml thiamine and 0.4% glucose were used for the cultivation of *E. coli* cells. Ampicillin (50 μg/ml), chloramphenicol (20 μg/ml), and/or spectinomycin (50 μg/ml) were added for selecting transformants and for growing plasmid-harboring cells.

### Strains, Plasmids, and Oligonucleotides

*Escherichia coli* K-12 strains, plasmids and oligonucleotides used in this work are listed in Supplementary Tables S1-S3, respectively. Construction of the individual strain and plasmids are described in Supplementary Material.

### Immunoblotting

Immunoblotting was carried out essentially as described previously (Akiyama et al., 2017). Protein samples were separated by SDS-PAGE and electroblotted onto an Immobilon-P membrane filter (MilliporeSigma). Proteins reacting with the indicated antibodies were visualized by Lumino image analyzer LAS-4000 mini (Cytiva) using ECL or ECL Prime Western Blotting Detection Reagents (Cytiva). In the substituted Cys accessibility analysis experiments, Immobilon-P^SQ^ membrane filter was used (MilliporeSigma). Anti-HA (HA-probe (Y-11), Santa Cruz Biotechnology), anti-Myc [c-Myc (9E10), Santa Cruz Biotechnology], rabbit polyclonal anti-RseP and anti-RseA antibodies (Hizukuri and Akiyama, 2012), anti-SecB (a gift from Shoji Mizushima’s Lab.) antibodies, and mouse monoclonal anti-Bla [Beta lactamase antibody GTX12251 (GeneTex Inc.)] antibodies were used for immunoblotting. Anti-HA and anti-SecB antibodies were pre-mixed and used to detect HA-tagged RseA-derivatives [HA-RseA or HA-MBP-RseA(LY1)148] and SecB simultaneously. Anti-Myc and anti-Bla antibodies were pre-mixed and used to detect RseP-His_6_-Myc and Bla simultaneously.

### *In vivo* Protease Activity Assay

The *in vivo* proteolytic activity of RseP was assayed as described previously (Akiyama et al., 2017) with slight modifications. Briefly, cells were grown at 30°C in the M9-based medium with 20 μg/ml each of the 20 amino acids for 3 h. Protein expression was induced for 3 h by adding 1 mM IPTG and 1 or 5 mM cAMP at the start of cultivation or for 30 min by adding them after 2.5 h cultivation. Proteins were precipitated by trichloroacetic acid (TCA) treatment and analyzed by SDS-PAGE and immunoblotting. Cleavage efficiencies of the substrates were calculated according to the following equation: Cleavage efficiency (%) = 100 × (cleaved)/[(cleaved) + (full length)], where (cleaved) and (full length) are intensities of the respective bands.

### Substituted Cysteine Accessibility Analysis

The -acetamide-4'-maleimidylstilbene-2,2'-disulfonic acid (AMS)-malPEG modification of substituted Cys residues was carried out essentially as described previously (Akiyama et al., 2017; Hizukuri et al., 2017). Spheroplasts were prepared from cells carrying a plasmid encoding a single-Cys derivative of RseP-HM by lysozyme/EDTA treatment, as described previously (Inaba et al., 2008), and treated with 1 mM (Thermo Fisher Scientific) in the presence or absence of 1% Triton X-100 at 24°C for 5 min. After incubation with 62.5 mM DTT at 24°C for 18 min to quench AMS, proteins were precipitated with 5% TCA and washed with 5% TCA and then with acetone. Samples were solubilized in 100 mM Tris-HCl (pH 8.1) containing 1% SDS and 1 mM Tris(2-carboxyethyl)phosphine (TCEP) by incubation at 37°C for 5 min. SDS-denatured proteins were treated with 5 mM methoxypolyethylene glycol 5,000 maleimide (malPEG; MilliporeSigma) at 37°C for 60 min with vigorous shaking to modify free thiols. AMS/malPEG modified proteins were analyzed by SDS-PAGE and anti-Myc immunoblotting. The proportion of a single Cys derivative of RseP-HM modified with AMS was calculated according to the following equation: AMS modification (%) = 100 × (*a* − *b*)/*a*, in which *a* is the ratio of the malPEG-modified forms to total RseP-HM in the control sample that are prepared without AMS treatment and *b* is the ratio of the malPEG-modified forms to total RseP-HM in the AMS-treated sample.

### *β*-Galactosidase Activity Assay

The σ^E^ activity was assayed by monitoring β-Galactosidase (LacZ) activity expressed from a chromosomal σ^E^-dependent *lacZ* reporter gene (*rpoH*P3-*lacZ*). Cells were grown at 30°C for 5 h in L medium supplemented with 0.1 mM IPTG and 1 mM cAMP with shaking in test tube. The LacZ activity of growing cells was measured essentially as described previously (Mori et al., 2018).

### Isolation of Deregulated RseP Mutants

Genetic screening for deregulated RseP mutants was carried out essentially as described previously (Inaba et al., 2008). Twenty independent mutagenized plasmid libraries were prepared by propagating plasmid pTM235 (encoding RseP-HM A326W mutant) for several generations at 37°C in mutator strains XL1-Red (Agilent) or KD1087 (Degnen and Cox, 1974). The library plasmids were introduced into TR71 carrying a chromosomal σ^E^-dependent *lacZ* reporter gene (*rpoH*P3-*lacZ*; Mecsas et al., 1993). Transformants were selected at 30°C on L agar plates containing 50 μg/ml ampicillin, 1 mM IPTG, 1 mM cAMP, 40 μg/ml 5-bromo-4-chloro-3-indolyl-*β*-D-galactopyranoside (X-gal), and 0.5 mM phenylethyl-*β*-D-thiogalactopyranoside (tPEG). Dark blue colonies were picked up, purified, and checked for their colony color by re-streaking on a X-gal-tPEG plate. Plasmid were prepared from the cells with dark blue color and subjected to DNA sequencing analysis.

### Site-Directed *in vivo* Photo Cross-Linking and Purification of Cross-Linked Products

Site-directed *in vivo* photo cross-linking was carried out essentially as described previously (Akiyama et al., 2017). Cells harboring pEVOL-pBpF and a plasmid encoding an RseP-HM derivative were grown at 30°C in M9 medium containing 0.5 mM *p*-benzoyl-L-phenylalanine (*p*BPA; Bachem AG) for 4 h. A portion of the culture was withdrawn and UV-irradiated for 10 min at 4°C by using B-100 AP UV lamp (365 nm; UVP, LLC.). In case of whole cell protein analysis, proteins were acid-precipitated, washed with acetone, and dissolved in 1 × SDS sample buffer. To purify cross-linked products, UV-irradiated cells were washed with and suspended in 30 mM Tris-HCl (pH 8.1) buffer and sonically disrupted on ice. After removal of unbroken cells by low-speed centrifugation, membranes were prepared by ultracentrifugation (100,000 × *g*, 60 min at 4°C) and solubilized with 1% *n*-dodecyl-*β*-D-maltoside in 50 mM Tris-HCl (pH 8.1) buffer. Cross-linked products containing hexahistidine-tagged RseP proteins were affinity-purified by binding to Ni-NTA agarose (Qiagen) and elution with 1× SDS sample buffer with 500 mM imidazole.

### Trypsin Susceptibility Assay

The trypsin susceptibility assay for RseP was performed essentially as described previously (Inaba et al., 2008). Cells carrying a plasmid encoding a derivative of RseP-HM were grown at 30°C in the M9-based medium supplemented with 20 μg/ml each of the 20 amino acids, 1 mM IPTG, and 1 mM cAMP for 3 h. Spheroplasts were prepared and treated with 2.5 μg/ml Trypsin on ice for the indicated time periods. A portion of the reaction solutions was mixed with an equal volume of 10% TCA, and acid-precipitated proteins were analyzed by SDS-PAGE and immunoblotting.

## Results

### Bacterial Group I S2P Peptidases Possess a PDZ Domain-Adjacent Periplasmic Region Containing a Predicted Amphiphilic Helix

**Figure 1:**
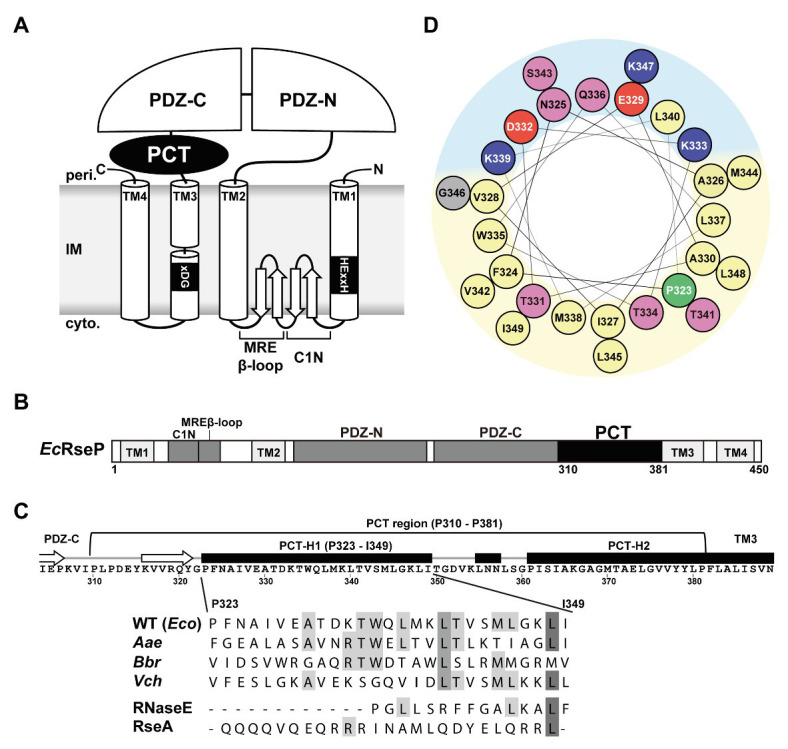
Features of the PDZ carboxyl terminal (PCT)-H1 segment of RseP. **(A)** Schematic representation of *Escherichia coli* RseP. Active site motifs (HExxH motif and xDG motif) are shown by black boxes. **(B)** Structural elements of RseP. The PCT region is shown in black. (**C**; Upper) The amino acid sequence and the PSI-PRED-predicted secondary structures around the PCT region. Regions predicted to form an α-helix and a β-strand are indicated by a black box and a white arrow, respectively. (Lower) Amino acid sequence alignment of the PCT-H1 segments of RseP homologs and the segment A of RNaseE and the hydrophilic helical region of RseA used in this study (*Eco*, *Escherichia coli*; *Aae*, *Aquifex aeolicus*; *Bbr*, *Bordetella bronchiseptica*; *Vch*, *Vibrio cholerae*). Amino acid sequences were aligned by the Jalview program (Waterhouse et al., 2009; http://www.jalview.org/). Conserved residues are boxed in gray. **(D)** Helical wheel representation of the PCT-H1 segment of *Ec*RseP produced by the HeliQuest program. Amino acids were colored as follows: positive, blue; negative, red; hydrophilic, pink; hydrophobic, yellow; proline, green; glycine, gray. Predicted hydrophilic and hydrophobic regions of the wheel were shown in light blue and light yellow, respectively.

Group I members of the S2P family peptidases possess a variable number of extracytoplasmic PDZ domains between the TMH2 and the TMH3 segments of the S2P core domain (Kinch et al., 2006). We discovered that the *E. coli* and most of the other bacterial Group I S2P homologs have a weakly-conserved sequence of approximately 70 amino acid residues between the periplasmic PDZ domain(s) and TMH3 (Figures 1A,B; Supplementary Figures S1A, S2). We named this region the PDZ carboxyl terminal (PCT) region. The PSI-PRED program (Jones, 1999)^1^ predicted that the PCT region of *E. coli* RseP (*Ec*RseP) contains two long α-helices (Figure 1C). We designated the N-terminal helix (Pro-323 to Ile-349) as the H1 segment and the C-terminal helix (Pro-361 to Pro-381) as the H2 segment. Analysis of the H1 segment with a helical wheel projection program HeliQuest (Gautier et al., 2008)^2^ revealed that this region can form a helix with strong amphiphilic properties (Figure 1D). Similarly, secondary structure analysis of 111 bacterial S2P homologs from a broad range of phyla (Supplementary Figure S2), such as *Aquifex aeolicus* (*Aa*RseP), *Bordetella bronchiseptica* (*Bb*RseP), and *Vibrio cholerae* (*Vc*RseP; Supplementary Figures S1A,B), indicated that their PCT regions contain multiple helices. The most N-terminal one (corresponding to H1) had a similar length (approximately 27 amino acid residues) and amphiphilic properties, while showing limited sequence conservation (Figure 1C; Supplementary Figure S1B). The predicted structural characteristics of the H1 segment raise the possibility that this segment plays an important role in the RseP function. Thus, we focused our further analyses on the H1 segment of *Ec*RseP.

### The H1 Segment Is Important for Both the Proteolytic Function and Protein Stability of RseP

**Figure 2:**
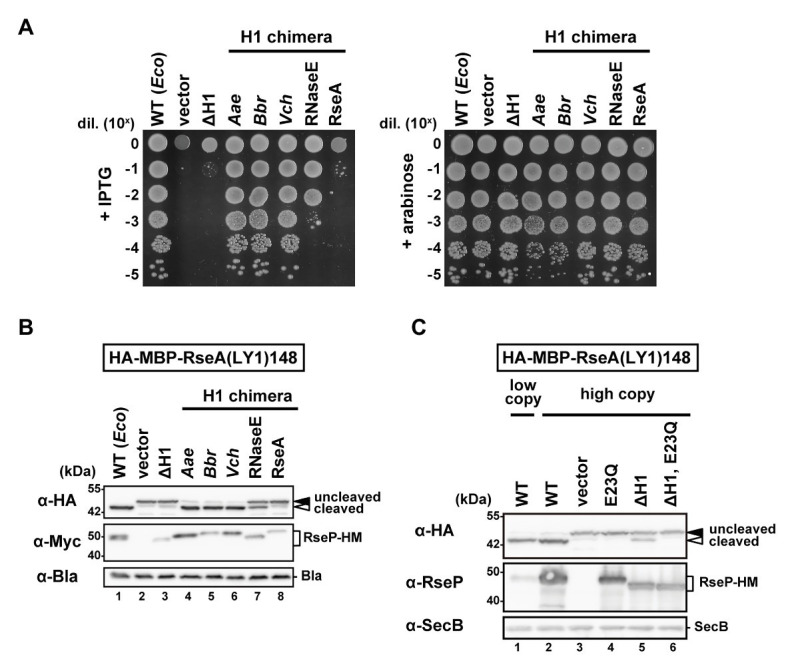
Complementation and proteolytic activities of the H1-deletion or H1-chimera mutants of RseP. **(A)** Growth complementation assay. KK31 [Δ*rseP*/pKK6 (P_BAD_-*rseP*)] cells carrying pKK11 (P_lac_-*Ec*RseP-HM, WT), pTWV228 (vector), pTM320 [P_lac_-RseP(ΔH1)-HM, ΔH1] or a plasmid encoding chimeric RseP-HM proteins in which the H1 of *Ec*RseP was replaced with the corresponding region of *Aa*RseP (*Aae*), *Bb*RseP (*Bbr*), and *Vc*RseP (*Vch*); the segment A of *Ec*RNaseE (RNaseE) or the periplasmic helix of *Ec*RseA (RseA) were grown in L medium containing 0.02% L-arabinose. Cultures were serially diluted with saline and spotted on L agar plates containing 1 mM IPTG (*left*, wild-type RseP or its derivatives was expressed from P_lac_) or 0.02% L-arabinose (*right*, wild-type RseP was expressed from P_BAD_). Plates were incubated at 37°C for 16.5 h. **(B)** Model substrate cleavage of RseP H1-deletion or H1-chimera mutants. KK211 (Δ*rseA* Δ*rseP*) cells harboring pYH20 (HA-MBP-RseA(LY1)148) were further transformed with pYH9 (RseP-HM), pSTD689 (vector), pTM324 (RseP(ΔH1)-HM) or a plasmid encoding the H1-chimera RseP-HM proteins as in **(A)**. Cells were grown at 30°C in M9-based medium containing 1 mM IPTG and 5 mM cAMP for 3 h. Proteins were analyzed by 10% Laemmli SDS-PAGE and anti-HA or anti-Myc/anti-Bla immunoblotting. *β*-lactamase expressed from plasmid serves as a loading control (α-Bla). *Uncleaved* and *cleaved* indicate the full-length and the RseP-cleaved forms of HA-MBP-RseA(LY1)148, respectively. **(C)** Substrate cleavage by the RseP H1-deletion mutant expressed from a pUC118-based high copy plasmid. KK211 cells harboring pYH20 were further transformed with pKK10 (RseP-HM, WT/low copy), pKK49 (WT/high copy), pUC118 (vector), pKA52 (E23Q, active site mutation), pTM470 (ΔH1) or pTM477 (ΔH1, E23Q). pMW118‐ and pUC118-derivatives were used as low-copy-number and high-copy-number plasmids, respectively. Protein samples were prepared as in **(B)** and analyzed by 10% Laemmli SDS-PAGE and anti-HA/anti-SecB or anti-RseP immunoblotting. Cytoplasmic protein SecB serves as a loading control (α-SecB). Positions of molecular size markers (in kDa) are shown on the left.

We first constructed an *Ec*RseP mutant lacking the H1 segment (ΔH1) by deleting the residues from Pro-323 to Ile-349 of RseP-HM (RseP with a C-terminal His_6_-Myc bipartite tag) and examined their growth complementation activity using an RseP-depletable strain (Kanehara et al., 2001). Expression of RseP(ΔH1)-HM mutant from a plasmid did not support the growth of an *rseP*-disrupted strain (Figure 2A). The *in vivo* proteolytic activity of the ΔH1 mutant was examined using a model substrate, HA-MBP-RseA(LY1)148 (Figure 2B). This model substrate, a derivative of a DegS-cleaved form of RseA (RseA148), has a haemagglutinin (HA)-tagged maltose-binding protein (MBP) domain and the first TM segment of lactose permease (LacY) in place of the cytoplasmic region and the TM segment of RseA148, respectively, and can be cleaved by RseP in a DegS-independent manner (Hizukuri and Akiyama, 2012). RseP-HM or its ΔH1 derivative was co-expressed with the model substrate in a Δ*rseA* Δ*rseP* cell (although *rseP* is an essential gene, it can be deleted in an *rseA* null background). The accumulation level of the ΔH1 mutant protein was markedly decreased compared to the accumulation level of the wild-type RseP-HM protein (Figure 2B, α-Myc), suggesting that the H1 deletion destabilized RseP. While the expression of the wild-type RseP-HM caused a nearly complete conversion of the substrate from the uncleaved form to the cleaved form, little conversion was observed with the ΔH1 mutant (Figure 2B, α-HA). Since the lack of the substrate cleavage upon expression of the ΔH1 mutant could be attributed to the decreased accumulation level of the mutant protein, we over-expressed the ΔH1 mutant using a high-copy number plasmid (Figure 2C). Expression of the wild-type or the ΔH1 mutant form of RseP using a high-copy number plasmid resulted in complete or partial conversion of the substrate, respectively (compare *lanes* 2 and 5). Derivatives of the wild-type or the ΔH1 mutant RseP carrying an additional protease active site mutation E23Q exhibited no substrate conversion, confirming that the observed conversion represented the RseP derivative-induced substrate cleavage (see *lanes* 4 and 6). While the accumulation level of the ΔH1 mutant protein expressed using the high-copy number plasmid was much higher than that of wild-type RseP expressed using the low-copy number plasmid, the ΔH1 mutant showed less efficient substrate cleavage, indicating that the H1 deletion affected both the stability and the proteolytic activity of RseP. These results suggest that the H1 segment is required for a stable and functional RseP structure.

We then examined the correlation between the amphiphilic nature of the H1 segment and its functions. For this, we constructed a chimeric RseP protein in which the H1 segment of *Ec*RseP had been replaced with either of the corresponding regions of *Aa*RseP, *Bb*RseP, or *Vc*RseP. As described above, while the H1 segments of *Ec*RseP and these three RseP homologs share low similarity in their amino acid sequences, they are all predicted to form an α-helix with strong amphiphilicity. These H1 chimeras exhibited full complementation activity (Figure 2A) against the *E. coli rseP-*disruption mutation and similar levels of model substrate cleavage to the original *Ec*RseP (Figure 2B). The protein accumulation levels of these chimeras were comparable to the *Ec*RseP levels (Figure 2B). Next, we replaced the H1 segment of *Ec*RseP with the segment A of *E. coli* RNaseE, which has been reported to form a 15-amino-acid amphiphilic helix (Khemici et al., 2008). Although the segment A of RNaseE is shorter than, and has no sequence similarity to, the H1 region, the segment A chimera accumulated at a similar level to the *Ec*RseP and showed significant growth complementation and substrate cleavage activities (Figures 2A,B). In contrast, a 25-amino acid hydrophilic helix of an unrelated amino acid sequence (a part of the periplasmic region of *E. coli* RseA) did not restore the stability and the proteolytic activity of the ΔH1 mutant, when replacing the H1 segment (Figure 2B). Overall, these results suggest that the amphiphilic property of the H1 segment is important for both the proteolytic function and protein stability of RseP.

### The H1 Segment of RseP Forms a Peripherally Membrane-Associated Helix

**Figure 3:**
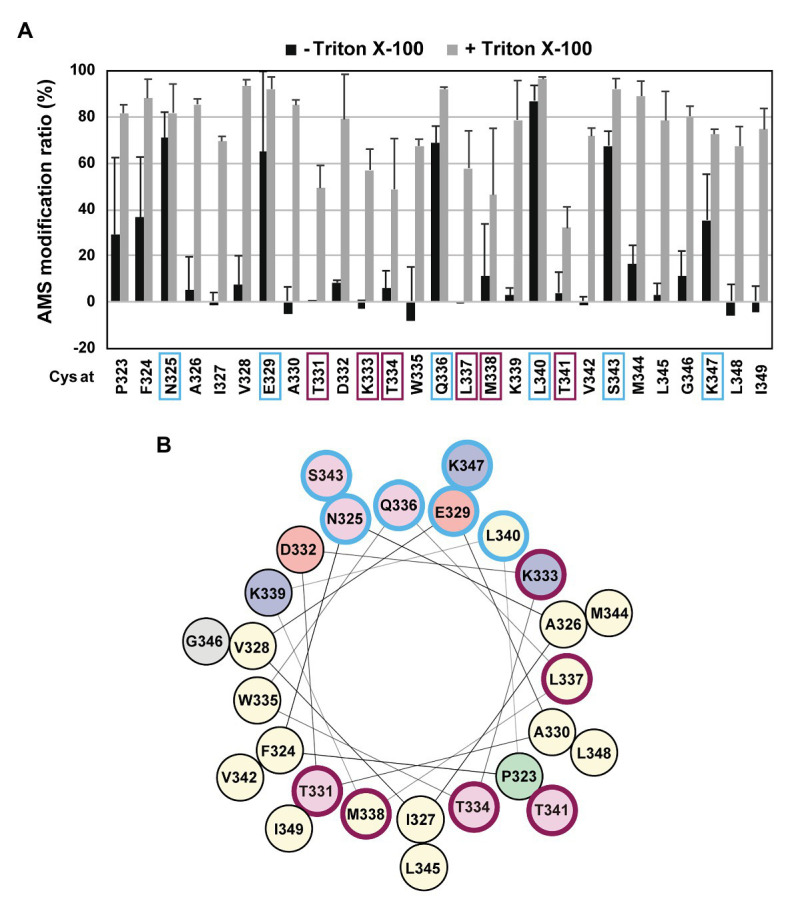
Substituted cysteine accessibility analysis of the H1 segment. **(A)** Spheroplasts prepared from KK374 (Δ*rseA* Δ*rseP* Δ*degS*) cells carrying a plasmid encoding a derivative of Cys-less RseP-HM possessing a single Cys residue at the indicated position (pTM101 derivatives) were treated with 1 mM 4-acetamide-4’-maleimidylstilbene-2,2’-disulfonic acid (AMS) in the presence or absence of 1% Triton X-100. After quenching AMS, proteins were precipitated with trichloroacetic acid (TCA), solubilized in 1% SDS, and treated with 5 mM malPEG. The samples were analyzed by 7.5% Laemmli SDS-PAGE and anti-Myc immunoblotting. The AMS modification ratio (%) in each condition is shown graphically (see Supplementary Figure S4 for the immunoblotting results). At least two independent experiments were carried out and the mean values are shown with standard deviations. **(B)** Helical wheel representation of the H1 segment described as in Figure 1D. In **(A,B)** residues at which substituted Cys showed high AMS modification ratio even without Triton X-100 treatment are circled in pale cyan and those at which substituted Cys showed low AMS modification ratio even after Triton X-100 treatment are circled in purple.

If the H1 region forms an amphiphilic helix, as predicted, it might be associated with the periplasmic surface of the membrane. To further acquire information on the H1 structure and its possible interaction with the membrane *in vivo*, we conducted the substituted cysteine accessibility analysis using AMS, a membrane-impermeable thiol-alkylating reagent. Previously, we successfully applied this method to investigate the mode of the membrane association and folding of the PDZ-tandem domain, the MRE β-loop and the C1N domains, and the active site region of RseP (Koide et al., 2007; Hizukuri et al., 2014; Akiyama et al., 2017). We introduced a Cys residue, at each position from Pro-323 to Ile-349 in the H1 segment of the Cys-less derivative (C33A/C427A) of RseP-HM. The Cys-introduced mutant proteins accumulated at a comparable level to the wild-type RseP and exhibited near-normal complementation and substrate cleavage activities (Supplementary Figures S3A,B), thus indicating that these Cys substitutions had little effect on the RseP’s structure and function. Spheroplasts were prepared from cells expressing the single-Cys derivative of RseP-HM and treated with AMS in the presence or absence of TritonX-100, a nonionic detergent (Figure 3; Supplementary Figure S4). The Cys residues in eight mutants (P323C, F324C, N325C, E329C, Q336C, L340C, S343C, and K347C) were efficiently modified with AMS even in the absence of the detergent, whereas the other Cys residues required membrane solubilization for substantial AMS-labeling (Figure 3A). Among them, the N325C, E329C, Q336C, L340C, S343C, and K347C residues were mapped on the hydrophilic side of the predicted amphiphilic helix (Figure 3, marked in pale cyan). These results support the notion that the H1 segment forms a partially membrane-embedded amphiphilic helical structure with its hydrophilic side exposed to the periplasmic surface. Note that the Pro-323 and Phe-324 were substantially modified with AMS in the absence of TritonX-100, although they are not expected to be located at the hydrophilic face of H1. As these residues are located at the N-terminal end of H1, they might not be involved in the stable formation of the H1 helix. Some residues showed relatively low AMS-induced modification even after the membrane structure was disrupted by treatment with Triton X-100 (Figure 3, marked in purple). These residues are located on the hydrophobic side of the predicted amphiphilic helix and clustered in the mid-region of H1. This region might be buried inside the RseP molecule or might interact tightly with other structural elements.

### The M338P Mutation in the H1 Segment Impairs the Proteolytic Function of RseP

**Figure 4:**
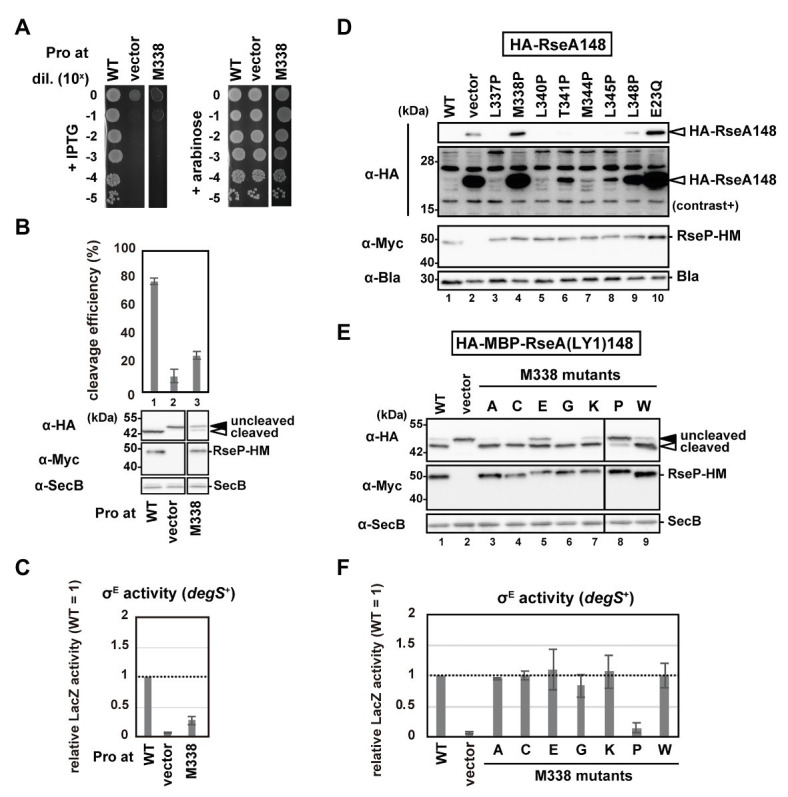
Systematic mutational analysis of the H1 segment. **(A)** Growth complementation assay of the RseP M338P mutant. KK31 [Δ*rseP*/pKK6 (P_BAD_-*rseP*)] cells carrying pKK11 (P_lac_-RseP-HM, WT), pTWV228 (vector) or a plasmid encoding an RseP-HM derivative were analyzed as in Figure 2A. Plates were incubated at 37°C for 19.5 h. **(B,E)** Model substrate cleavage by RseP mutants with an amino acid substitution at Met-338. KK211 (Δ*rseA* Δ*rseP*) cells harboring pYH20 (HA-MBP-RseA(LY1)148) were further transformed with pYH9 (RseP-HM), pSTD689 (vector) or a plasmid encoding RseP-HM derivatives with the indicated mutation. Cells were grown at 30°C in M9-based medium for 2.5 h and additionally incubated with 1 mM IPTG and 5 mM cAMP for 0.5 h, and analyzed as in Figure 2. Cleavage efficiencies were calculated as the ratio of the cleaved form to the total proteins of HA-MBP-RseA(LY1)148 and graphically represented in **(B)**. At least two independent experiments were carried out and the mean values are shown with standard deviations. **(C,F)** σ^E^ activity of M338 RseP mutants in the *degS^+^* background. Cells of *rpoH*P3*-lacZ* reporter strain AD2469 (*degS*^+^ Δ*rseP*) harboring pSTD343 (*lacI*) were further transformed with pKK11 (RseP-HM, WT), pTWV228 (vector) or a plasmid encoding RseP-HM derivatives with the indicated substitution. Cells were grown at 30°C in L medium containing 0.1 mM IPTG and 1 mM cAMP for 5 h and LacZ activity was measured. Calculated values were normalized by that of the strain expressing wild type RseP (WT = 1). At least two independent experiments were carried out and the mean values are shown with standard deviations. **(D)** Cleavage of HA-RseA148 by RseP mutants with a Pro substitution in H1. AD1840 (Δ*rseA* Δ*rseP* Δ*degS*) cells harboring pTM685 (HA-RseA148) were further transformed with pKK11 (RseP-HM), pTWV228 (vector) or a plasmid encoding an RseP-HM derivative. Cells were grown at 30°C in M9-based medium containing 1 mM IPTG and 1 mM cAMP for 3 h, and analyzed as in Figure 2B. The second panel from the top is a signal-enhanced image of the top panel (contrast+).

The Cys substitution mutants (Supplementary Figure S3) indicate that none of the H1 residues are essential for the RseP function, which is consistent with the results obtained from chimeric RseP-HM derivatives with the H1 region from other species or the unrelated amphiphilic helix of RNaseE (Figure 2). Thus, we performed a proline-scanning mutagenesis against the H1 region (Phe-324 to Ile-349), since Pro substitutions could locally alter or destabilize the helical structure of H1. Complementation and model substrate cleavage assays using HA-MBP-RseA(LY1)148 showed that the Pro mutants, except M338P, exhibited roughly normal activities (Figures 4A,B; Supplementary Figures S5A,B). In contrast, the M338P mutant was defective both in complementation and in substrate cleavage. All the Pro mutants including M338P accumulated normally (Figure 4B; Supplementary Figure S5B). These results demonstrated that the M338P mutation severely affected the proteolytic function of RseP.

Next, we examined the σ^E^ activity in the cells expressing the Pro mutants using a reporter gene (*rpoH*P3-*lacZ*) in which *lacZ* is placed under the σ^E^-dependent promoter *rpoH*P3 (Figure 4C; Supplementary Figure S5C), as it will reflect the ability of the RseP mutants to cleave chromosomally-encoded RseA. This reporter gene enables quantitative evaluation of the cellular σ^E^ activity (Mecsas et al., 1993; Hizukuri and Akiyama, 2012). We expressed the Pro mutants in a Δ*ompA* Δ*ompC* Δ*rseP* strain carrying the *rpoH*P3-*lacZ* reporter gene (*rseP* can be disrupted in a strain lacking the two outer membrane proteins, OmpA and OmpC; Douchin et al., 2006), and analyzed the LacZ activity. The wild-type RseP-expressing strain showed a low but significantly higher level of the σ^E^ activity than the vector control strain (Figure 4C; Supplementary Figure S5C, compare WT and vector). This “basal-level” σ^E^ activation would represent a constant cleavage of RseA by DegS at a low level even under normal (low stress) growth conditions, leading to the subsequent RseP-catalyzed cleavage of the DegS-cleaved RseA and the final σ^E^ activation (Alba et al., 2001; Lima et al., 2013). While the cells expressing the Pro mutants other than M338P showed nearly the same σ^E^ activity as the wild-type RseP-expressing cells, the M338P mutant-expressing cells showed a significantly lower σ^E^ activity (Figure 4C; Supplementary Figure S5C), suggesting that the M338P mutant protein is defective in RseA cleavage. To further confirm the effects of the Pro mutations on the RseA cleavage, we expressed the wild-type RseP and several Pro mutants including M338P with HA-RseA148, an N-terminally HA-tagged RseA derivative mimicking the DegS-cleaved intermediate form of RseA. Immunoblotting analysis using anti-HA antibody showed that cells expressing the M338P mutant accumulated HA-RseA148 at similar levels to the cells expressing the proteolytically-inactive E23Q mutant and the vector control cells (Figure 4D, *lanes* 2, 4, 10). Collectively, these results suggest that the RseP M338P mutant exhibits an impaired RseA cleavage function.

To further investigate the underlying mechanism through which the M338P mutation affects the RseP function, we replaced Met-338 with several other amino acid residues having a side-chain of a different size and chemical property and examined the ability of the resulting mutants to cleave HA-MBP-RseA(LY1)148 and activate σ^E^ (Figures 4E,F). The results showed that the mutations other than M338P were mostly silent, although the glutamate substitution slightly affected the model substrate cleavage. M338 is positioned in the middle of the hydrophobic face of the H1 helix, and it has been previously shown in a membrane-mimicking environment that among the 20 amino acids, proline, and glutamate have the first and second lowest helix-forming propensities, respectively (Liu and Deber, 1998). These results suggest that destabilization of the H1 helix around Met-338 impairs the substrate cleavage by RseP *in vivo*.

### The H1 Helix Is Involved in the PDZ Domain-Mediated Substrate Discrimination

Stress signals induce two successive cleavages of RseA, first within its periplasmic region by DegS and second within its TM segment by RseP. In this process, the PDZ tandem of RseP acts as a size-exclusion filter that only allows for cleavage of periplasmically-processed form of, but not full-length, RseA by RseP (Inaba et al., 2008; Hizukuri and Akiyama, 2012; Hizukuri et al., 2014). Previously we isolated a variety of RseP mutations which caused a deregulated cleavage of RseA, that is, a DegS-independent cleavage of full-length RseA, by RseP. While most of the mutations were mapped in the PDZ domains, some occurred outside of these domains. A326V is one of the latter class of mutations and is located in the N-terminal part of the H1 helix (Inaba et al., 2008). Thus, H1 might be implicated in the PDZ-mediated regulation of substrate cleavage. We therefore addressed this possibility.

**Figure 5:**
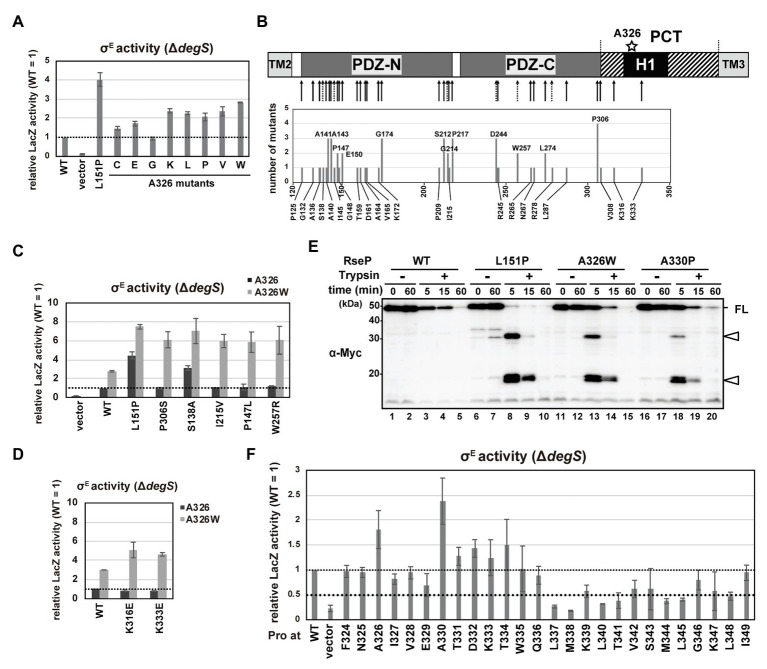
DegS-independent cleavage of full-length RseA by the RseP H1 mutants. **(A,C,D,F)** DegS-independent σ^E^ activation by the RseP H1 mutants. Cells of *rpoH*P3*-lacZ* reporter strain AD2473 (Δ*degS* Δ*rseP*) harboring pSTD343 (*lacI*) were further transformed with pKK11 (RseP-HM, WT), pTWV228 (vector), or a plasmid encoding the indicated RseP-HM derivatives. LacZ activity was measured as in Figure 4C. At least two independent experiments were carried out and the mean values are shown with standard deviations. **(C,D)** Activities of wild-type RseP or the RseP mutants carrying the indicated mutant with (gray columns) or without (black columns) A326W mutation. **(B)** Schematic representation of the second-site mutation sites of the *rseP* mutants that were isolated by random mutagenesis of the *rseP(A326W)* plasmid. The positions in RseP at which mutations were newly isolated in this work (solid arrow) or those at which deregulation mutations were previously isolated (Inaba et al., 2008; dashed arrow) are shown in the upper part. Numbers of independently-isolated mutants at the same position were shown in the lower graph. **(E)** Trypsin susceptibility of deregulated RseP mutants. Spheroplasts prepared from KK374 (Δ*rseA* Δ*rseP* Δ*degS*) cells carrying pKK11 (RseP-HM, WT) or a plasmid encoding an RseP-HM derivative were incubated at 0°C with or without 2.5 μg/ml Trypsin for the indicated periods. TCA-precipitated proteins were analyzed by 7.5% Laemmli SDS-PAGE and anti-Myc immunoblotting. FL indicates the intact RseP-HM protein. Open arrowheads indicate tryptic fragments of RseP.

The DegS-independent cleavage of RseA by RseP was evaluated by measuring the σ^E^ activity in the Δ*degS* strain using the *rpoH*P3-*lacZ* reporter (Figure 5A). As previously reported, cells expressing the L151P mutant, one of the strongest deregulated RseP mutants with the mutation in the PDZ-N domain (Inaba et al., 2008), exhibited approximately 4 times higher LacZ activity than cells expressing the wild-type RseP (Figure 5A). We constructed several RseP mutants with various amino acids substitutions for Ala-326 and tested their ability to activate σ^E^. Cells expressing most of the mutants exhibited an increased σ^E^ activity compared to the wild-type RseP, indicating that the mutation of Ala-326 induces DegS-independent cleavage of RseA by RseP. We assumed that the H1 helix functions co-operatively with other regions of RseP including the PDZ domains to regulate the sequential cleavage of RseA and tried to isolate a mutation that synergistically increases the cleavage of full-length RseA in a Δ*degS* background when combined with the A326W mutation (the Ala-326 mutation with the most noticeable effect). To accomplish this, a plasmid carrying the *rseP(A326W)* mutant gene mutagenized in a mutator strain to obtain randomly mutated plasmid libraries. Next, we introduced the plasmid libraries into a *rpoH*P3-*lacZ* reporter strain and searched for dark blue colonies on plates containing 5-bromo-4-chloro-3-indolyl-*β*-D-galactopyranoside (X-gal), a chromogenic substrate of LacZ. We picked up 150 dark blue colonies out of the approximately 5.6 × 10^4^ transformants and finally obtained 41 different mutants at 34 positions in the plasmid-borne *rseP* gene (Figure 5B; Supplementary Figure S6A). Several identical mutations were obtained from independently prepared libraries, suggesting that the mutations were nearly saturated. Thirty-one of the 34 mutation sites were found in the PDZ tandem. They included the mutations that occurred at the same amino acid positions as those we obtained in the previous experiments in which a wild-type RseP plasmid was used for preparation of the mutagenized libraries; some mutations were identical to the previously isolated ones (4 mutations), but the others caused synonymous (1 mutation) or non-synonymous (3 mutations) codon changes. In addition, two mutations (K316E and K333E) were obtained in the PCT region; one (K316E) was in a predicted β-strand upstream of H1 while the other (K333E) was in the mid-region of H1, and one mutation was in a loop region N-terminal to PDZ-N.

We first characterized the mutations in the PDZ domains. When mapped on the PDZ tandem structure, a considerable number of mutations were located around the core region of PDZ-N or in the capping helix that covers the putative ligand-binding groove of PDZ-N (Supplementary Figure S6B). We selected five mutations (S138A, P147L, and I215V in the PDZ-N core region, and W257R and P306S in the PDZ-C core region) and examined their individual effects on the substrate-discriminating function of the PDZ tandem as a single mutation or in combination with A326W by using a Δ*degS* strain carrying the σ^E^ reporter gene. Expression of the double mutant forms of RseP carrying one of the above mutations in addition to A326W elevated the LacZ activity 6–8-fold compared to the wild-type RseP (Figure 5C). In contrast, RseP carrying a single mutation (either of the five aforementioned mutations or A326W) did not increase (P147L, I215V, W257R, and P306S) or slightly increased (S138A and A326W) LacZ activity. These results indicate that the A326W mutation exhibits a synergistic effect on the PDZ function when combined with either of the P147L, I215V, W257R, and P306S mutations. Consistently, the RseA degradation assay showed that the double mutants degraded full-length RseA in the a Δ*degS* background more efficiently than the single mutants (Supplementary Figure S6C). No or low synergistic effects were observed in the reporter assay for the combination of A326W with the S138A or L151P mutation (Figure 5C), suggesting that specific combinations of mutations are required for a high synergistic effect. These results indicate a functional interaction between the H1 helix and the PDZ tandem. While the newly-isolated PCT mutations, K316E and K333E, exerted hardly detectable effects on σ^E^ activation as single mutations, they substantially increased the σ^E^ activity when combined with A326W, supporting the involvement of the PCT region in the PDZ functionality (Figure 5D).

Previous studies showed that deregulated RseP mutants having PDZ-N mutations including the L151P mutation exhibited increased trypsin susceptibility (Inaba et al., 2008; Hizukuri et al., 2014). This might be explained by the mutation-induced structural disorder (Hizukuri et al., 2014), which leads to generation of discrete degradation products when spheroplasts expressing these mutant RseP proteins were treated with trypsin. To investigate whether the H1 mutations also induce a similar structural change in the PDZ domains, we examined the trypsin susceptibility of the RseP(A326W) mutant (Figure 5E). The wild-type RseP was slowly degraded by trypsin but generated no detectable amount of degradation fragment. In contrast, the L151P mutants showed an elevated trypsin susceptibility and generated several tryptic fragments after 5‐ or 15-min of trypsin treatment. The A326W mutant showed a trypsin degradation profile similar to the L151P mutant. This result strongly suggests that the A326W mutation in H1 induced a structural change in the PDZ tandem similar to the L151P mutation in the PDZ-N. This further supports the idea that the PDZ domains and the H1 helix are structurally and functionally interrelated.

Additionally, we examined the DegS-independent RseA-cleavage activity of the systematically-constructed proline mutants of the RseP H1 segment previously described (Figure 5F). Cells expressing several RseP derivatives carrying a Pro mutation in the N-terminal half of the H1 helix showed slightly or substantially higher σ^E^ activity than cells expressing the wild-type RseP. In particular, the A330P mutant elevated the σ^E^ activity to a higher level than the A326P mutant. The A330P mutant showed a similar tryptic fragment pattern to the L151P and A326W mutants, suggesting that the A330P mutation might induce a specific structural alteration in the PDZ tandem (Figure 5E). In contrast, a considerable number of Pro mutations in the C-terminal half of the H1 helix decreased the σ^E^ activating ability of RseP. Regardless of the effects on σ^E^ activation, all Pro mutants except M338P retained the ability to cleave HA-MBP-RseA(LY1)148 model substrate (Figure 4B; Supplementary Figure S5B). We further examined the proteolysis of HA-RseA148 by seven mutants (L337P, M338P, L340P, T341P, M344P, L345P, and L348P) that exhibited a 0.5-fold or less σ^E^ activity compared to the wild-type RseP and found that most of them retained the near normal protease activity against HA-RseA148, although the L348P mutant degraded HA-RseA148 with slightly lower efficiency (Figure 4D). Thus, the decreased σ^E^ activation associated with these mutants was not ascribed to their impaired protease activity. These results further support the involvement of H1 in maintaining the proper substrate-discriminating function of the PDZ domains.

### The H1 Helix Interacts With the DegS-Cleaved Form of RseA

**Figure 6:**
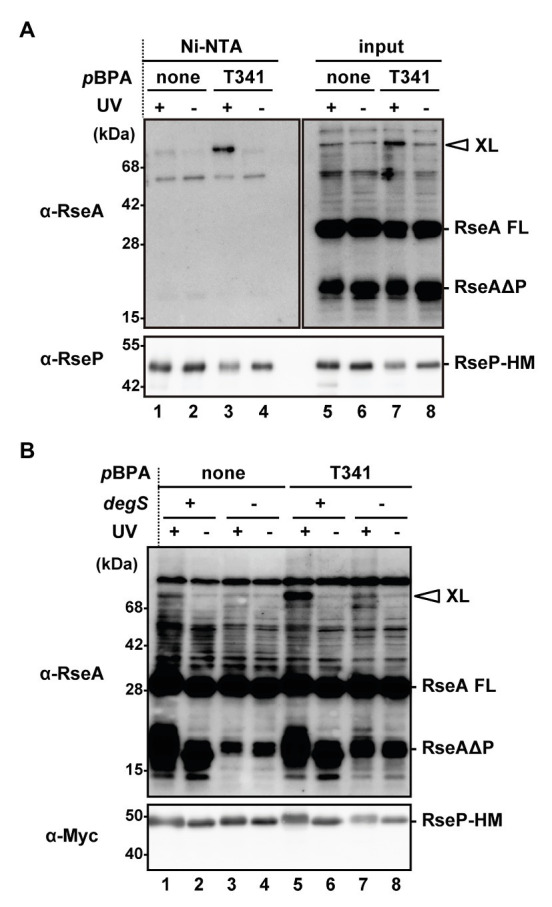
*In vivo* photo-crosslinking between RseP H1 and RseA. **(A)** Purification of the cross-linked product of RseP T341 *p*BPA mutant. KA418 (Δ*rseP*) cells harboring pEVOL-pBpF were further transformed with a plasmid encoding RseP(E23Q)-HM with (pTM375) or without (pKA52) T341 amber mutation. Cells were grown at 30°C in M9-based medium supplemented with 0.5 mM *p*BPA for 4 h and UV-irradiated for 10 min (UV+) or not (UV−). Membrane fractions were isolated, solubilized with 1% DDM, and subjected to Ni-NTA affinity-purification. TCA-precipitated total proteins after UV-irradiation (input) and affinity-purified proteins (Ni-NTA) were analyzed by 12.5% (anti-RseA) or 7.5% (anti-RseP) Laemmli SDS-PAGE and immunoblotting. Proteins from approximately nine-fold more cells were loaded on the gel for the purified samples compared with the input samples. XL indicates the cross-linked product between RseP-HM and chromosomally-expressed RseA. RseA FL and RseAΔP indicate the full-length and the DegS-cleaved form of RseA, respectively. **(B)** DegS dependent cross-linking of the RseP(T341*p*BPA) mutant with RseAΔP. KA418 (*degS*^+^ Δ*rseP*) or KA438 (Δ*degS* Δ*rseP*) cells harboring pEVOL-pBpF were further transformed with pKA52 or pTM375. Cells were grown and UV-irradiated as in **(A)** and then whole cell lysates were analyzed by 12.5% (anti-RseA) or 7.5% (anti-Myc) Laemmli SDS-PAGE and immunoblotting. Note that the band with a similar size to RseAΔP in the Δ*degS* strain was presumably a DegS-independent degradation product of RseA (Kanehara et al., 2003).

Based on the aforementioned results, we hypothesized that a physical interaction might be present between the H1 segment and the PDZ domains. Furthermore, since the PCT region is connected to the TM3 that has been shown to interact with RseA, H1 might interact with a substrate as well. To assess the possible intra‐ and inter-molecular interactions, we performed a site-directed *in vivo* photo-cross-linking experiment targeting the H1 segment. Previously, this technique enabled us to detect the interaction of several RseP regions with RseA (Akiyama et al., 2015, 2017). The *p*-benzoylphenylalanine (*p*BPA), a non-natural photoreactive amino acid analog (Young et al., 2010), was systematically introduced at each position between Pro-323 and Ile-349, of the H1 helix of RseP-HM using amber suppression. We used a Δ*ompA* Δ*ompC* Δ*rseP* strain carrying the wild-type chromosomal *rseA* gene to detect possible interaction of H1 with chromosomally encoded RseA. When the RseP-HM derivatives with *p*BPA in H1 were expressed, the accumulation levels of the DegS-cleaved form of RseA (RseAΔP) were clearly decreased, indicating that they retained a substantial proteolytic activity (Supplementary Figure S7). For the photo-crosslinking experiment, we introduced an active site mutation (E23Q) into the *p*BPA-incorporated RseP derivatives to prevent substrate degradation by RseP during the experiments. Cells expressing RseP(*p*BPA) were UV-irradiated and whole cell proteins were analyzed by immunoblotting (Supplementary Figure S8). Upon UV-irradiation, an RseP-HM derivative having *p*BPA at the Tyr-69 position in the MRE β-loop generated an approximately 71 kDa cross-linked product with RseA that was detected with an anti-RseA antibody, as previously reported (Akiyama et al., 2015). We found that RseP(T341*p*BPA) reproducibly generated anti-RseA-reactive band of a similar size in an UV irradiation-dependent manner (Supplementary Figure S8). To verify this band as an RseP-RseA cross-linked product, we purified the cross-linked product containing RseP-HM by His-tag affinity isolation (Figure 6A). The approximately 71 kDa product, which was generated in an UV irradiation‐ and *p*BPA-dependent manner, was His-tag affinity-isolated and detected with an anti-RseA antibody, indicating that it was an RseP-RseA crosslinked product. The generation of this cross-linked product was dependent on the presence of chromosomally encoded DegS (Figure 6B), thus suggesting that it was a cross-linked product between RseP-HM and RseAΔP. These results suggest that the H1 helix can directly interact with RseAΔP. We failed to detect any distinct cross-linked bands that would represent intra-molecular cross-linking in RseP.

## Discussion

Site-2 proteases are involved in regulation of a wide variety of cellular processes thorough cleavage of specific substrates. However, the mechanism of the substrate discrimination and recognition remains obscure. Bacterial S2P homologs with one or more PDZ domains, such as *E. coli* RseP, exhibit a PCT periplasmic region of approximately 70 amino acid residues of unknown functions downstream of the PDZ domain(s) (Supplementary Figure S2). This region commonly contains an H1 segment predicted to form an amphiphilic helix. In this study, we focused on the H1 segment of *E. coli* RseP and investigated its structure and function, from the viewpoint of its possible involvement especially in the substrate discrimination and recognition. The substituted Cys-accessibility analysis demonstrated that the H1 segment of RseP indeed assumes an amphiphilic helical structure partially buried in the membrane. The domain substitution and scanning mutagenesis analyses suggested that the helical and amphiphilic properties, but not specific amino acid residues or the length, of the H1 helix are important for the structural stability and proteolytic function of RseP. Furthermore, the results of the *in vivo* cross-linking experiments showed that the H1 helix is in close proximity to a substrate, suggesting that H1 directly contacts the substrate. Moreover, we showed that several mutations in H1, including A326W, affected the PDZ-mediated substrate discrimination and altered the conformation of the PDZ domains, similar to the previously isolated PDZ mutations. In addition, the effect of the A326W mutation in H1 on the PDZ function was synergistically amplified when combined with several PDZ mutations. These results suggest that the H1 helix cooperates with the PDZ domains in substrate recognition of RseP.

**Figure 7:**
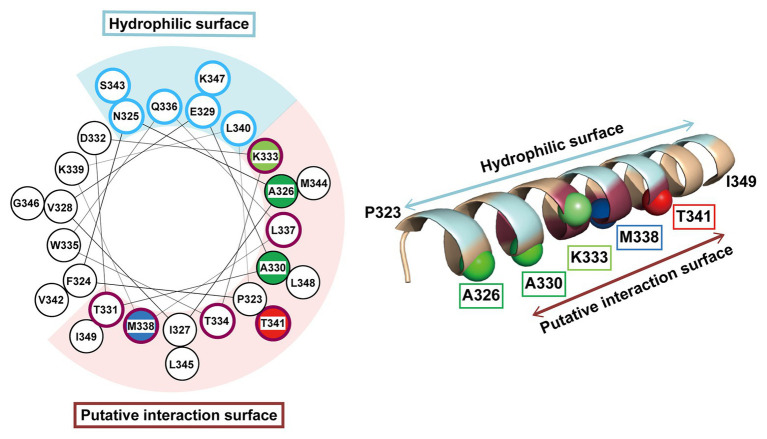
Mapping of the characteristic residues on the helical structure of H1. The characteristic residues and regions revealed by this study are mapped on a helical wheel representation (left) or a 3D α-helix model (right) of H1. The residues are colored and represented by sphere (right) as follows: blue, the residue whose Pro substitution impaired the proteolytic function of RseP; red, the residue that was photo-cross-linked with RseA; green, the residues whose Pro substitution caused deregulated cleavage of RseA; light green, the residue whose Pro substitution enhanced the deregulated RseA cleavage when combined with A326W. The solvent-exposed residues and the residues putatively involved in intra‐ or inter-molecular interaction (based on the results of Cys-accessibility analysis) were circled (left) and colored (right) in pale cyan and purple, respectively.

The *p*BPA introduced at the Thr-341 position within the H1 segment was cross-linked to the DegS-processed RseA. Thus, it is likely that H1 directly interacts with the substrate around this position. However, mutations of Thr-341 caused little defects in the substrate cleavage activity of RseP. This suggest that Thr-341 does not play an essential role in the interaction with the substrate. Thr-341 might form a substrate binding site together with other residues located in its vicinity, in which the individual residues provide limited contribution to the interaction with the substrate. While such a site might not act in stable substrate binding, it might enable substrate positioning for further processing. Intriguingly, the Pro substitution of a nearby residue, Met-338, severely impaired the substrate cleavage (Figure 4). Thr-341 and Met-338 are located adjacently on the same face of the predicted H1 helix structure (Figure 7). Met-338 might also contribute to the formation of the putative substrate binding site, and its substitution with Pro might interfere with this process by inducing an H1 conformational change. In contrast, other Pro substitutions (e.g., Ala-330 or Lys-333) little affected the RseP activity. Since H1 is predicted to form a long, membrane-associating helix, it may be rather robust against a helix destabilizing mutation and a Pro substitution may only locally affect the H1 structure. As a result, a Pro substitution located distantly from Met-338 may not significantly impair the possible substrate binding mediated by Met-338.

Previous structural and biochemical studies strongly suggest that the protease active site of RseP is located within the membrane (Feng et al., 2007; Koide et al., 2008; Akiyama et al., 2015, 2017). We have previously proposed a sequential substrate binding model in which, after passing through the PDZ-filter, the substrate first interacts with the partially-membrane-embedded region (C1N) of RseP on the cytoplasmic side of the membrane, and then it is transferred to the intramembrane β-hairpin like structure (MRE β-loop) which allows the presentation of the substrate to the catalytic site (Akiyama et al., 2015, 2017). In addition to C1N and the MRE β-loop, the periplasmically-oriented N-terminal part of TM3 has been shown to interact with the substrate (Koide et al., 2008). Since TM3 has an Asp residue that acts as a ligand for the catalytic zinc ion, it could bind a substrate that has been accommodated in the active site. The PCT region is located just upstream of TM3, thus H1 might cooperate with TM3 in the substrate binding process. In this respect, the putative substrate binding region around Thr-341 of H1 can be regarded as an exosite (sub-binding site) located on the periplasmic side. The presence of exosites that promote and regulate substrate cleavage has also been suggested in other IMPs such as γ-secretase (Kornilova et al., 2005; Fukumori and Steiner, 2016) and rhomboid (Strisovsky et al., 2009; Arutyunova et al., 2014; Cho et al., 2016; Shokhen and Albeck, 2017).

The substituted Cys accessibility analysis showed that at some positions within the H1 segment, Cys was inefficiently modified with AMS even after the membrane structure was disrupted by treatment with a nonionic detergent. Most of these positions are located on the hydrophobic side of the predicted amphiphilic helix and cluster in the mid-region of H1 (Figure 3B, purple). The inefficient AMS-modification of the aforementioned sites might reflect the interaction between the hydrophobic face of the H1 helix and the membrane domain of RseP. DegS would be another candidate for interaction, because a previous genetic analysis suggested its interaction with RseP (Grigorova et al., 2004), although no direct evidence for the interaction has been provided. Interestingly, although among the aforementioned sites, Thr-341 was the least AMS-accessible residue in the presence of Triton X-100, and *p*BPA at this position showed significant cross-linking with RseA. If the low AMS modifiability at Thr-341comes from the localization of this residue inside the folded RseP or interaction with other structural elements, the region around Thr-341 might undergo some conformational change in the process of recognition and binding of a substrate. We did not detect any intra-molecular photo-crosslinking between H1 and other parts of RseP including the PDZ domain. It cannot be excluded that this apparent lack of crosslinking resulted from some technical problems. For example, since the *p*BPA-photo-crosslinking approach would be sensitive to the orientation of the *p*BPA side chain, not all the components in close proximity to *p*BPA might be crosslinked (Miyazaki et al., 2020). Also, it would be possible that intra-molecularly-crosslinked RseP and uncrosslinked RseP cannot be separated on SDS-PAGE as intra-molecular crosslinking could cause a very slight mobility shift. Further studies including exhaustive chemical cross-linking are required to investigate these intra‐ or inter-molecular interactions.

While the current study demonstrated the structural and functional importance of the membrane-surface amphiphilic helix (H1) in *E. coli* RseP, and likely other bacterial S2Ps, some of the other families of IMPs have an element(s) with a similar structure and function. For instance, it has been shown that human presenilin 1 (PS1) has a partially-membrane-embedded α-helical structural region in the hydrophilic loop 1 (HL1) located in the extracellular/luminal side of the membrane (Sun et al., 2015; Takagi-Niidome et al., 2015). HL1 forms a binding site for a juxtamembrane side of the Aβ protein, together with the C-terminal region of PS1, which contributes to the Aβ cleavage at the ε‐ and γ‐ positions (Takagi-Niidome et al., 2015). A molecular dynamics simulation study (Lee et al., 2017) suggested that HL1 stabilizes the closed form of the γ-secretase complex by interacting with Nicastrin, a subunit of the γ-secretase with a large extra-membrane domain that serves as a molecular gatekeeper which blocks substrates with large ectodomains from interacting with γ-secretase (Bolduc et al., 2016), similar to the PDZ tandem of RseP. Bacterial rhomboid family proteins including *E. coli* GlpG have an amphiphilic loop structure (L1) containing multiple short helices between the first and the second TM segments. The L1 of *E. coli* GlpG is located on the periplasmic (extracytoplasmic) surface of the membrane (Wang et al., 2006; Maegawa et al., 2007). It was shown to contribute to the structural stabilization of GlpG by forming multiple intra-molecular hydrogen bonds (Baker and Urban, 2012) and participate in the formation of the substrate-binding pocket (Zoll et al., 2014). These observations indicate that a membrane-surface amphiphilic structure might be important for the stability and/or substrate recognition of different IMP families. Interestingly, a recent study suggested that the amphiphilic property of the GlpG L1 loop might have an important contribution to the distortion of the lipid structure around GlpG, which would facilitate diffusion of the enzyme in the membrane (Kreutzberger et al., 2019), raising the possibility that the membrane-surface amphiphilic helixes have some additional roles in the functions of IMPs.

**Figure 8:**
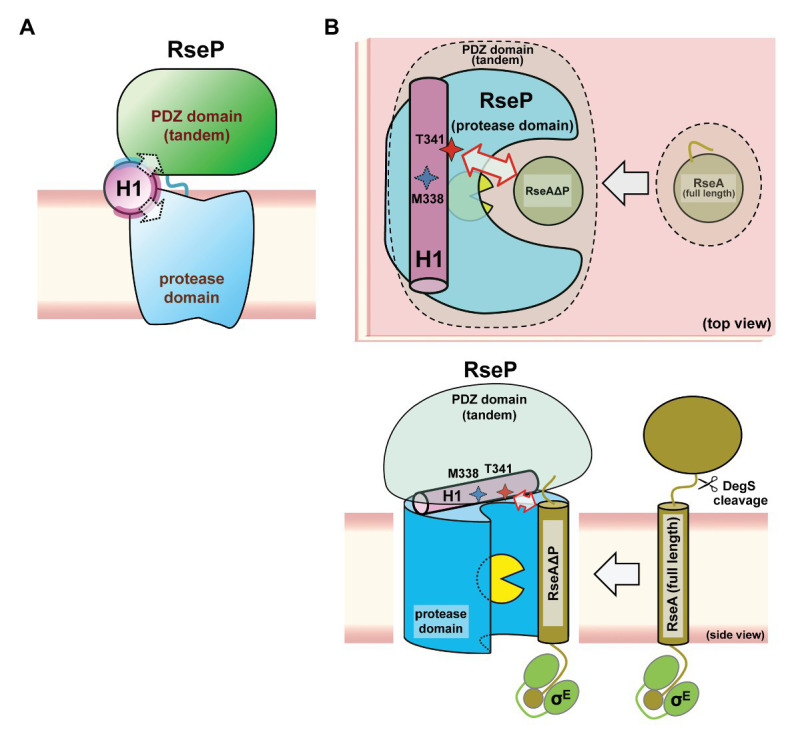
A models of the PCT-H1 helix as an adaptor linking the PDZ tandem and the protease domain of RseP. **(A)** An expected position of the H1 in RseP. H1 links the PDZ tandem and the protease domain. The solvent-exposed surface and the putative interaction surface (see Figure 7) are shown in pale cyan and purple, respectively. **(B)** Possible roles of H1 in the PDZ function and the substrate binding. H1 could be required for the PDZ tandem to be positioned properly to function as a size-exclusion filter, DegS-cleaved form of RseA (RseAΔP) gains access to the active site (yellow packman) through the PDZ filter. There, H1 could interact with the substrate to facilitate its efficient cleavage. A region around Thr-341 residue of H1 forms an exosite. (Upper) Top view: perpendicular to the membrane plane from the periplasmic side. (Lower) Side view: parallel to the membrane plane.

Our genetic and biochemical evidence suggest a functional correlation between the PDZ tandem and the H1 segment. Here, we showed that most PDZ mutations and H1 mutations in its N-terminal half elevated the σ^E^ activity in the Δ*degS* strain by increasing the DegS-independent cleavage of full-length RseA by RseP. On the other hand, several mutations in the C-terminal half of H1 decreased the σ^E^ activity, suggesting that they repressed the DegS-independent cleavage of full-length RseA. Thus, the H1 mutations can either positively or negatively influence the substrate-discriminating function of the PDZ domains. It would be conceivable that H1 directly interacts with the PDZ domains, and modulates, when mutated, their size-exclusion filter function by altering their configuration on the protease domain. Some of the H1 residues (Ala-326, Ala-330, and Lys-333) whose Pro substitution caused deregulated RseA cleavage are mapped on the same face of the predicted α-helical structure of H1 (Figure 7). Furthermore, Met-338 and Thr-341, which might be involved in the interaction with the substrate, are also located on the same face of the α-helical structure. This face is in good agreement with the one that is speculated to interact with some other cellular factors including other parts of RseP from the AMS modification assay (Figure 7, purple residue). We propose that the H1 helix is positioned between the membrane-embedded protease domain and the periplasmic PDZ tandem and directly interacts with these domains as an adaptor that connects these domains structurally and functionally, although we do not provide direct experimental evidence demonstrating the physical interaction of H1 with the PDZ and the protease domains of RseP (Figure 8). In addition, H1 might provide a site for substrate binding required for proper positioning and efficient cleavage. Deletion of H1 would alter relative dispositions of the PDZ domains against the protease domain, leading to the destabilization of the whole protein.

To verify the adaptor model described above, the determination of the RseP’s structure alone and as a complex with the substrate and a detailed analysis of the intra-molecular disposition of the PCT region and the H1 helix in the membrane-associated functional RseP are required. Furthermore, it would be interesting to investigate whether the H1 helix in other Group 1 bacterial S2P homologs with varying number of PDZ domains also have similar functions to RseP and/or some species-specific functions. Understanding the roles of the other parts in the PCT region awaits future study. In addition, we expect clarifying the mode and the timing of H1-substrate interaction in the substrate recognition and cleavage process to be essential for elucidating the underlying mechanism of intramembrane cleavage by RseP.

## Data Availability Statement

The original contributions presented in the study are included in the article/Supplementary Material, further inquiries can be directed to the corresponding authors.

## Author Contributions

YH and YA conceived the idea and supervised the study. TM, YH, and YA designed the experiments, analyzed the data, and wrote the manuscript. TM and YH performed the experiments. All authors contributed to the article and approved the submitted version.

### Conflict of Interest

The authors declare that the research was conducted in the absence of any commercial or financial relationships that could be construed as a potential conflict of interest.

## Footnotes

^1^http://bioinf.cs.ucl.ac.uk/psipred/

^2^https://heliquest.ipmc.cnrs.fr/
