# Supplementary material for: Involvement of a Membrane-Bound Amphiphilic Helix in Substrate Discrimination and Binding by an Escherichia coli S2P Peptidase RseP
Source: Front Microbiol. 2020 Nov 27;11:607381. doi: 10.3389/fmicb.2020.607381 (PMC7728848; doi:10.3389/fmicb.2020.607381)
Supplement: Supplementary file 1 [file Data_Sheet_1.pdf]

# *Supplementary Material*

## 1 Supplementary Figures

- Figure S1 ~ S8

## 2 Supplementary Methods

- Strains, plasmids and oligonucleotides

## 3 Supplementary Tables

- Table S1. *E. coli* strains
- Table S2. Plasmids
- Table S3. Primers and oligonucleotides

## 4 References

# Supplementary Figures

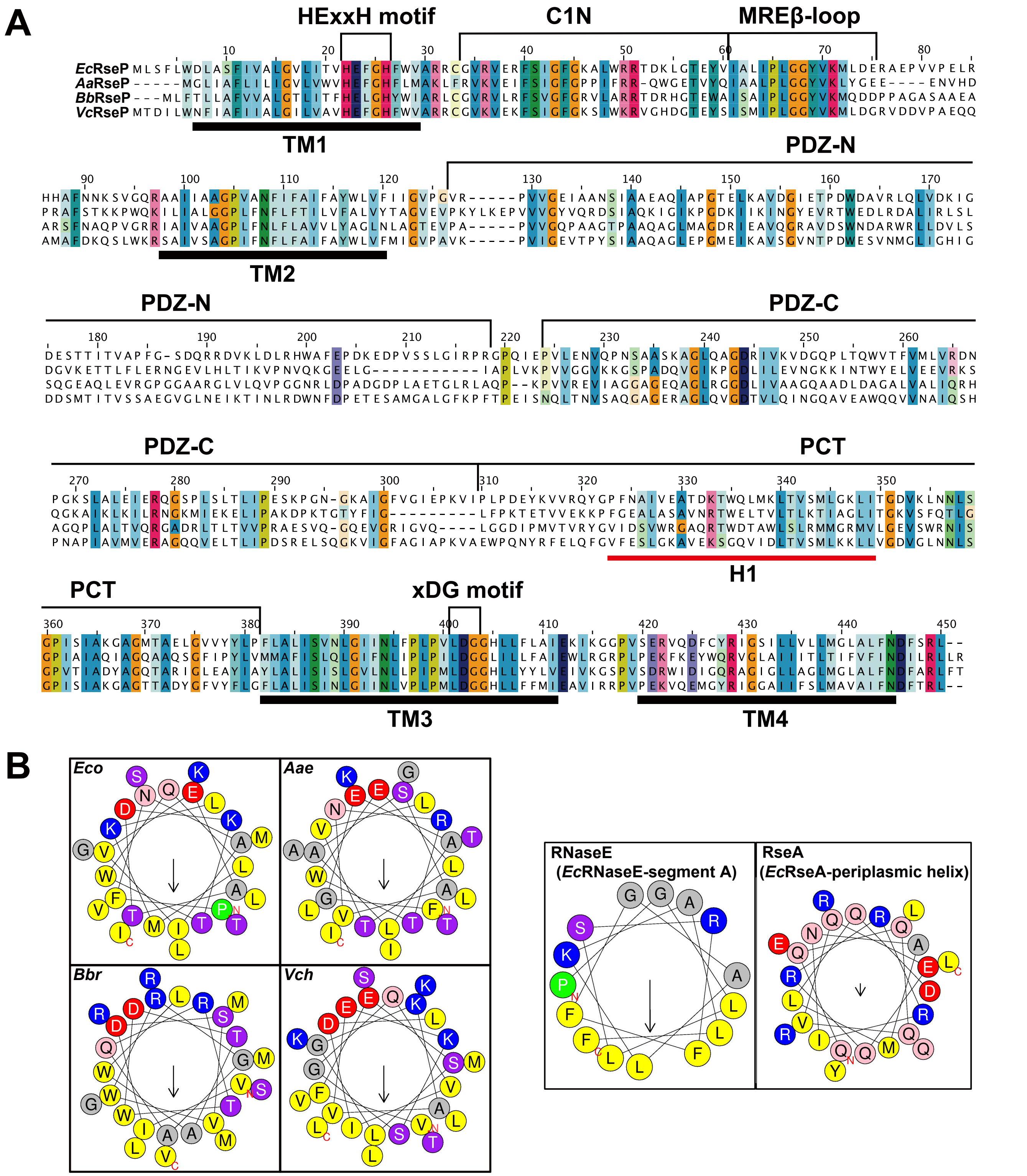


**Supplementary Figure S1. Comparison of bacterial RseP homologs and their predicted PCT-H1 segments. (A)** Amino acid sequence alignment of the four RseP orthologues used in this study. *Ec*RseP, RseP of *E. coli* (UniProtKB accession number: P0AEH1); *Aa*RseP, RseP orthologue from *A. aeolicus* (UniProtKB: O67776); *Bb*RseP, from *B. bronchiseptica* (UniProtKB: A0A0H3LMM6); *Vc*RseP, from *V. cholerae* (UniProtKB: Q9KPV9). Sequences were aligned by Jalview (http://www.jalview.org/). Conserved and similar residues are shown by colored boxes. Predicted TM segments, domains, and structural elements are based on that of *Ec*RseP. **(B)** Helical wheel representations of the predicted PCT-H1 segments of the RseP orthologues shown above. The helical structures were generated by HeliQuest (https://heliquest.ipmc.cnrs.fr/). RNaseE, the segment A of *Ec*RNaseE (UniProtKB: P21513); RseA, the hydrophilic helix of the periplasmic region of *Ec*RseA (UniProtKB: P0AFX7). Arrows indicate the direction and relative magnitude of the hydrophobic moment of each helix. N and C in red indicate the N- and C-terminal residue of each sequence, respectively.

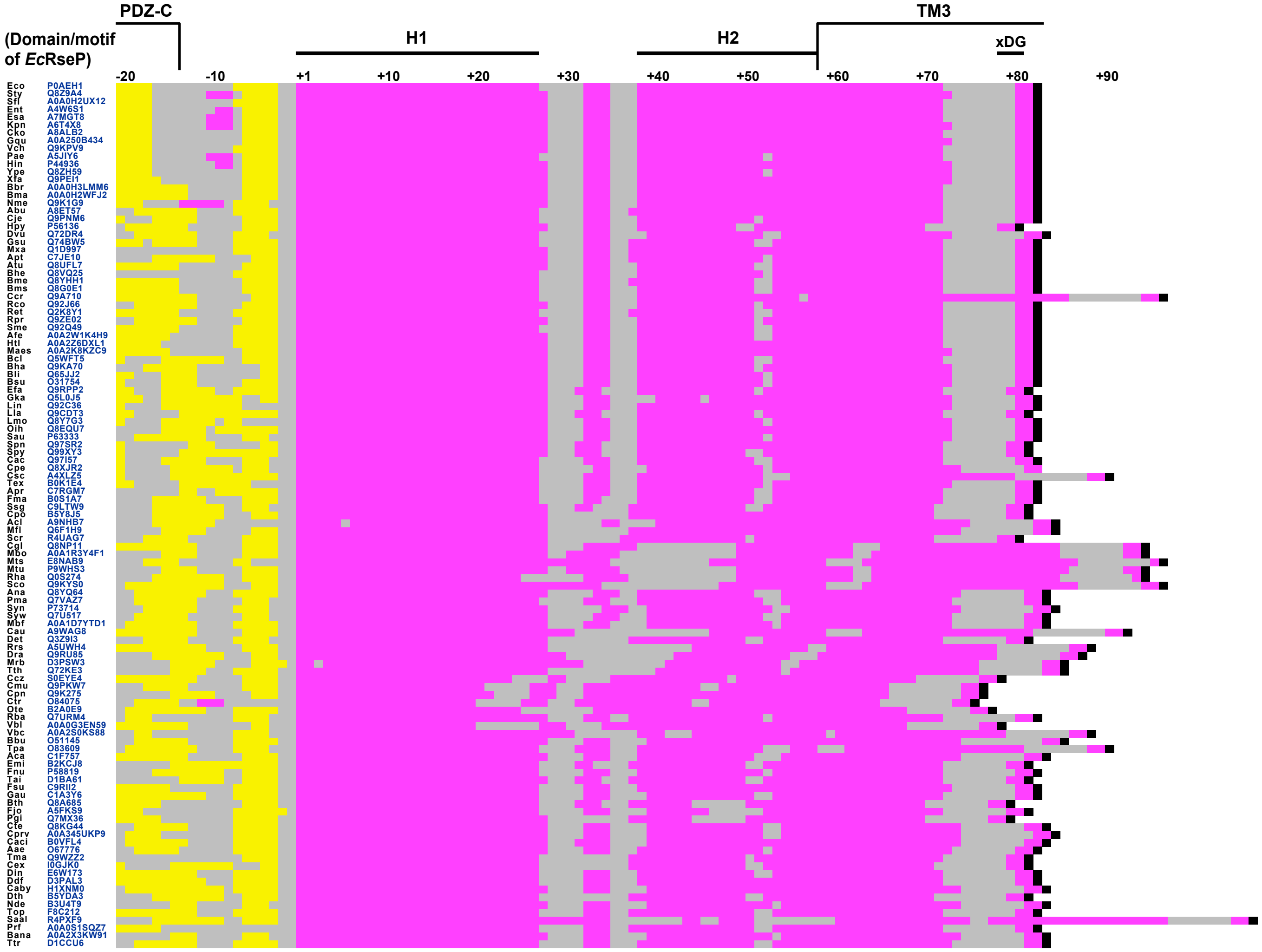


**Supplementary Figure S2. Alignment of the predicted secondary structural elements around the PCT region.** Secondary structural elements around the PCT regions from 111 bacterial Group I S2P homologues of a broad range of phyla were predicted by the PSI-PRED server and aligned. Candidate sequences of S2P homologues were collected as M50 family zinc-metallopeptidases using DBGET search (https://www.genome.jp/dbget/) against Pfam database (El-Gebali et al., 2019) with 'Peptidase_M50' motif. Detected sequences were manually checked to find canonically-conserved LDGG sequence in TMH3 of M50 family zinc metallopeptidases and the upstream ~100 a.a. regions were analyzed by the PSI-PRED program (http://bioinf.cs.ucl.ac.uk/psipred/). The secondary structures in the PCT regions were aligned by assigning the first residue of the predicted H1 segments to the +1 position. Alignment from the -20 position to the LDGG sequence were displayed. UniprotKB accession number of each sequence are indicated in the left side. Pink, α-helix; yellow, β-strand; gray, coil; black, the C-terminally adjacent residue of the canonically-conserved LDGG sequence. Domains and motifs of *E. coli* RseP are shown above the alignment.

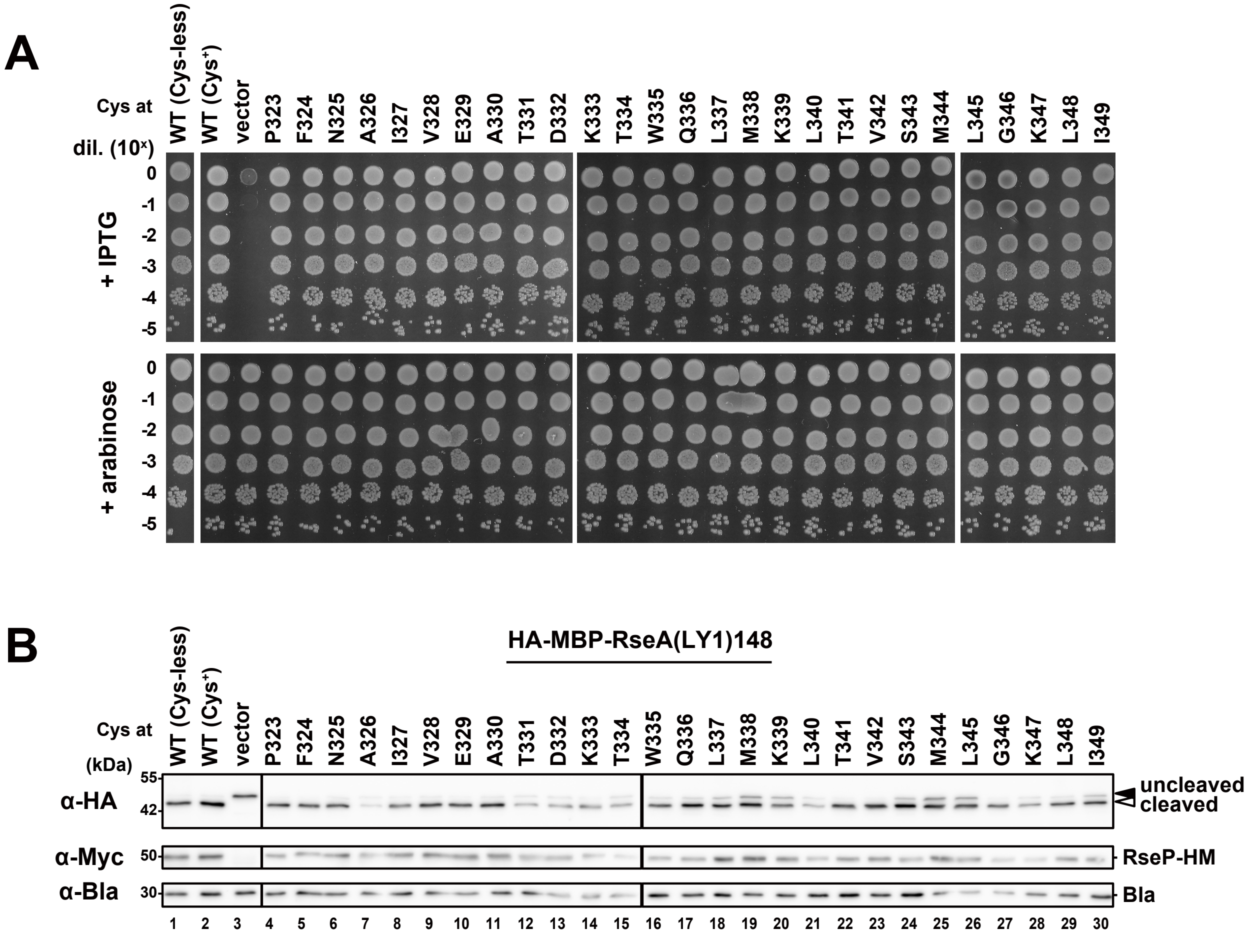


**Supplementary Figure S3. Cysteine-scanning mutagenesis analysis of the H1 segment. (A)** Growth complementation assay of single Cys RseP mutants. KK31 (Δ*rseP*/pKK6 ($P_{BAD}$-*rseP*)) cells carrying pTM101 (RseP(Cys-less)-HM), pKK11 (RseP-HM, $Cys^{+}$), pTWV228 (vector) or a plasmid encoding a derivative of RseP-HM with a unique Cys at the indicated position were grown in L medium containing 0.02% L-arabinose. Cultures were serially diluted with saline and spotted on L agar plates containing 1 mM IPTG or 0.02% L-arabinose. Plates were incubated at 37°C for 14.5 h. **(B)** Model substrate cleavage by single Cys RseP mutants. KK211 (Δ*rseA* Δ*rseP*) cells harboring pYH20 (HA-MBP-RseA(LY1)148) were transformed with pTM101 (RseP(Cys-less)-HM), pYH9 (RseP-HM, $Cys^{+}$), pSTD689 (vector) or a plasmid encoding single Cys RseP-HM derivatives. Cells were grown at 30°C in M9-based medium containing 1 mM IPTG and 5 mM cAMP for 3 h. Proteins were analyzed by 10% Laemmli SDS-PAGE and anti-HA or anti-Myc/anti-Bla immunoblotting. *β*-lactamase expressed from plasmid serves as a loading control (α-Bla).

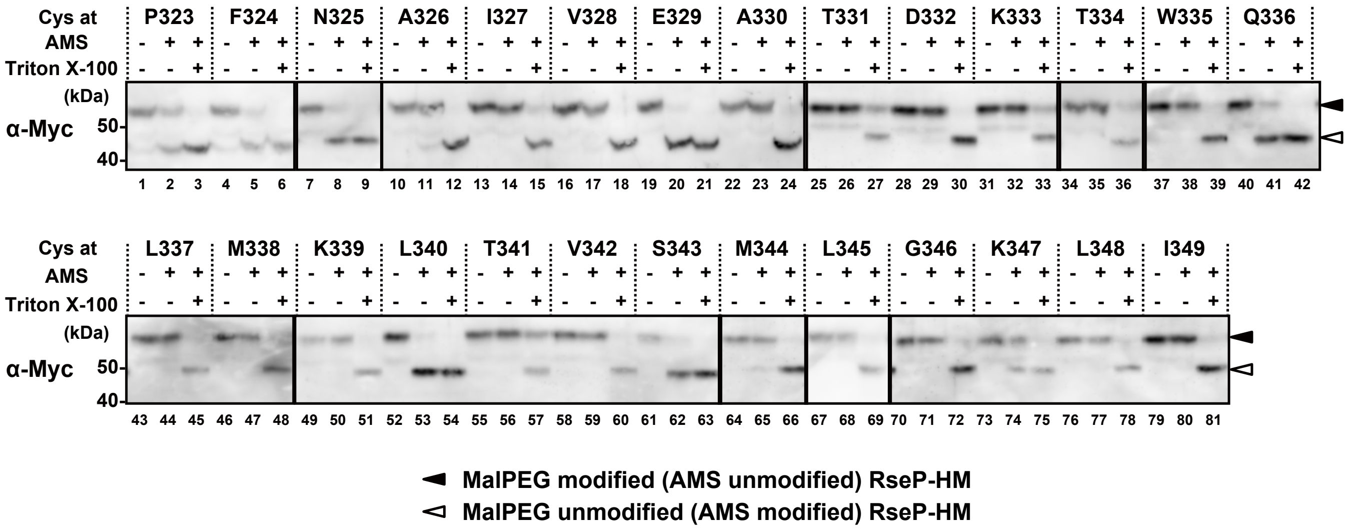


**Supplementary Figure S4. Substituted cysteine accessibility analysis of the H1 segment.** Spheroplasts prepared from KK374 (Δ*rseA* Δ*rseP* Δ*degS*) cells carrying a plasmid encoding a derivative of RseP-HM possessing a unique Cys residue at the indicated position (pTM101 derivatives) were treated with 1 mM AMS in the presence or absence of 1% Triton X-100. After quenching AMS, proteins were precipitated with TCA, solubilized in 1% SDS, and treated with 5 mM malPEG. The samples were analyzed by 7.5% Laemmli SDS-PAGE and anti-Myc immunoblotting. Filled and open arrowheads indicate malPEG-modified (*i.e.* AMS-unmodified) and malPEG-unmodified (AMS-modified) RseP, respectively. At least two independent experiments were carried out and the AMS modification ratio (%) were calculated and shown graphically in Figure 3.

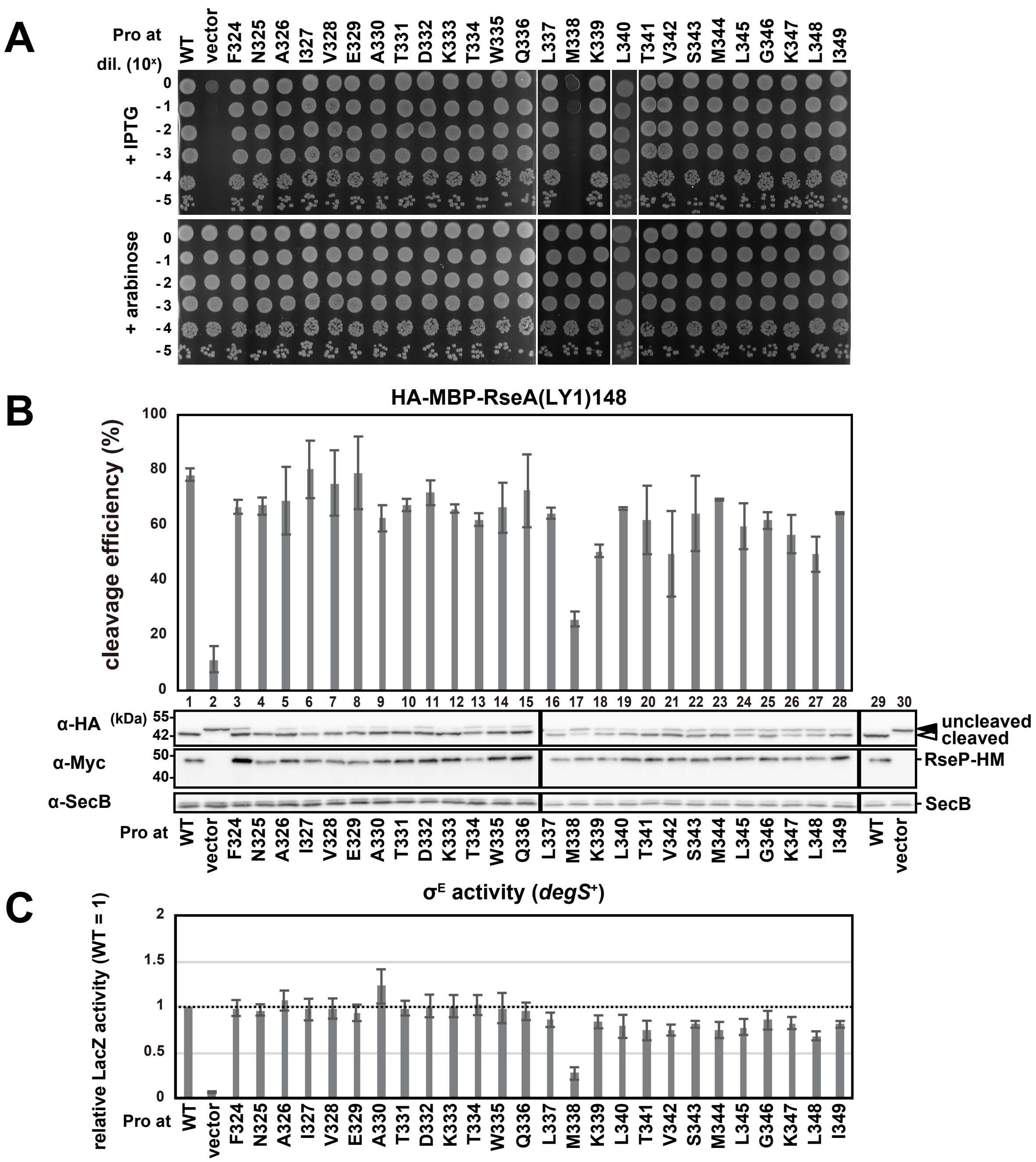


**Supplementary Figure S5. Proline-scanning mutagenesis analysis of the H1 segment. (A)** Growth complementation assay of Pro-substituted RseP mutants. KK31 (Δ*rseP*/pKK6 ($P_{BAD}$-*rseP*)) cells carrying pKK11 (RseP-HM, WT), pTWV228 (vector) or a plasmid encoding RseP-HM derivatives with a Pro-substitution at the indicated potion in H1 were grown in L medium containing 0.02% L-arabinose. Cultures were serially diluted with saline and spotted on L agar plates containing 1 mM IPTG or 0.02% L-arabinose. Plates were incubated at 37°C for 19.5 h. **(B)** Model substrate cleavage by RseP Pro mutants. KK211 (Δ*rseA* Δ*rseP*) cells harboring pYH20 (HA-MBP-RseA(LY1)148) were transformed with pYH9 (RseP-HM), pSTD689 (vector) or a plasmid encoding Pro-substituted RseP-HM derivatives. Cells were grown at 30°C in M9-based medium for 2.5 h and additionally incubated with 1 mM IPTG and 5 mM cAMP for 0.5 h. Proteins were analyzed by 10% Laemmli SDS-PAGE and anti-HA or anti-Myc/anti-SecB immunoblotting. SecB serves as a loading control (α-SecB). Sample sets (*lane 1 - lane 15*) and (*lane 16 - lane 30*) were electrophoresed separately. Cleavage efficiencies were calculated as the ratio of cleaved form to total proteins of HA-MBP-RseA(LY1)148 and graphically represented. At least two independent experiments were carried out and the mean values are shown with standard deviations. **(C)** DegS-dependent $\sigma^E$ activity of cells expressing RseP Pro mutants. Cells of *rpoH*P3-*lacZ* reporter strain AD2469 (*degS*+ Δ*rseP*) harboring pSTD343 (*lacI*) were transformed with pKK11 (RseP-HM, WT), pTWV228 (vector) or a plasmid encoding Pro-substituted RseP-HM derivatives. Cells were grown at 30°C in L medium containing 0.1 mM IPTG and 1 mM cAMP for 5 h and LacZ activity was measured. Calculated values were normalized by that of the strain expressing wild type RseP (WT = 1). At least two independent experiments were carried out and the mean values are shown with standard deviations. **(A, B, C)** The data of M338P mutant from the Pro-scanned mutants were shown in Figure 4A, B, C, respectively.

**A**

| mutation | Number of isolates | domain/ segment |
|---|---|---|
| P125L | 1 | loop |
| G132S | 1 | PDZ-N |
| A136Y | 1 | |
| S138A* | 1 | |
| A140W | 1 | |
| A141V | 1 | |
| A141Y | 2 | |
| A143E | 1 | |
| A143V | 2 | |
| I145N* | 1 | |
| P147L | 2 | |
| G148V | 1 | |
| E150K | 2 | |
| T159S | 1 | |
| D161G | 1 | |
| A164Y | 1 | |
| V165E | 1 | |
| K172E | 1 | |
| G174E | 1 | |
| G174S | 2 | |
| P209S | 1 | |
| S212L | 2 | |
| G214R* | 1 | |
| I215V | 1 | |
| P217L | 1 | |
| P217S | 2 | |
| D244G* | 1 | PDZ-C |
| D244N*** | 1 | |
| D244Y*** | 1 | |
| R245G | 1 | |
| W257R** | 2 | |
| R265W | 1 | |
| N267D | 1 | |
| L274S | 2 | |
| R278K*** | 1 | |
| L287S | 1 | |
| P306S | 2 | |
| P306T | 2 | |
| V308G | 1 | |
| K316E | 1 | PCT (β-strand) |
| K333E | 1 | PCT-H1 |

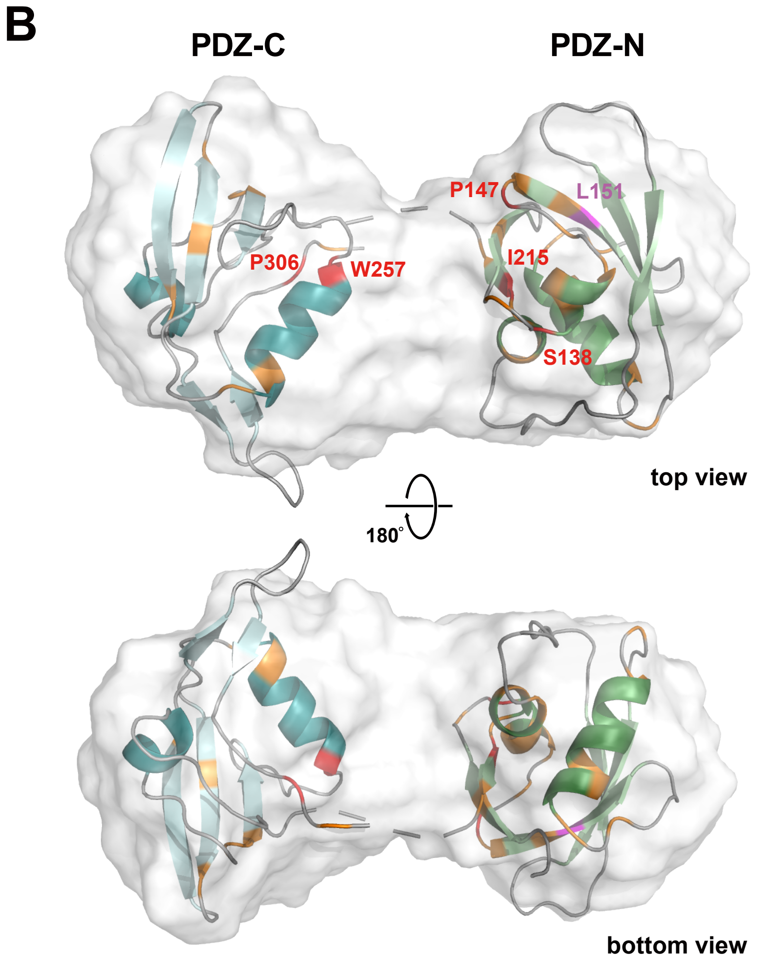


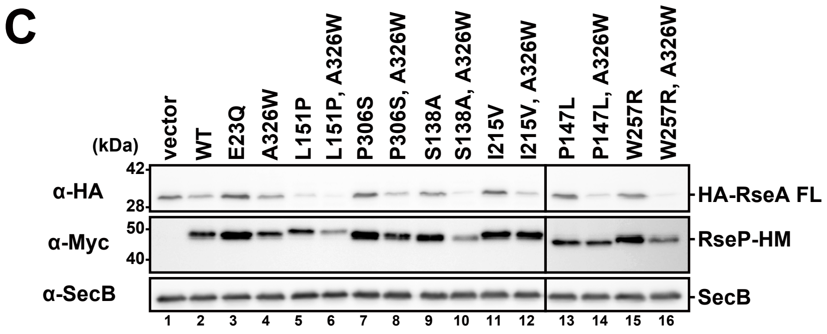


**Supplementary Figure S6. Mutations isolated by the random mutagenesis and screening. (A)** RseP mutants that caused the increased DegS-independent $\sigma^E$ activation were screened from the randomly mutagenized libraries of pTM235 carrying rseP(A326W). Numbers of the isolates of the second-site mutations and the domain/segment affected by the mutations were shown in the table. The mutations were marked as follows: identical (*), synonymous (**), or non-synonymous (***) mutations to those isolated in the previous screening (Inaba et al., 2008). **(B)** Mapping of the isolated mutations on the structure model of the PDZ-tandem of *E. coli* RseP (Hizukuri et al., 2014) in which the crystal structures of *Ec*PDZ-N and -C domains were superimposed on the envelope of the dummy-residue model from SAXS analysis. Ribbon representation of PDZ-N and -C domain is shown in green and cyan, respectively. The positions of the isolated mutations were shown in orange, and those analyzed in this study were shown in red. Position of Leu-151 is shown in magenta. Top view represents a view of the membrane plane from the periplasmic side, and bottom view from the cytoplasmic side. **(C)** DegS-independent cleavage of the full-length RseA. AD1840 (Δ*rseA* Δ*rseP* Δ*degS*) cells harboring pSTD691 (HA-RseA) were transformed with pKK11 (RseP-HM, WT), pTWV228 (vector), pKK34 (E23Q) or a plasmid encoding RseP-HM mutants with or without A326W mutation. Cells were grown at 30°C in M9-based medium containing 1 mM IPTG and 1 mM cAMP for 3 h. Proteins were analyzed by 12.5% (anti-HA/anti-SecB) or 10% (anti-Myc) Laemmli SDS-PAGE and immunoblotting. SecB serves as a loading control.

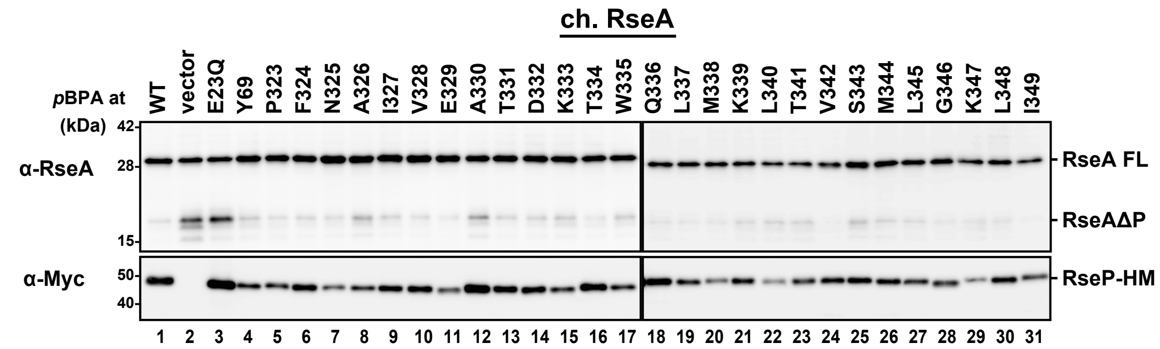


**Supplementary Figure S7. Protease function of the pBPA-substituted RseP mutants.** KA418 (Δ*rseP rseA*+) cells harboring pEVOL-pBpF were transformed with pKK49 (RseP-HM, WT), pUC118 (vector), pKA52 (E23Q) or a plasmid encoding RseP-HM with an amber mutation at the indicated position. Cells were grown at 30°C in M9-based medium supplemented with 0.5 mM *p*BPA for 4 h. Proteins were analyzed by 12.5% (anti-RseA) or 10% (anti-Myc) Laemmli SDS-PAGE and immunoblotting.

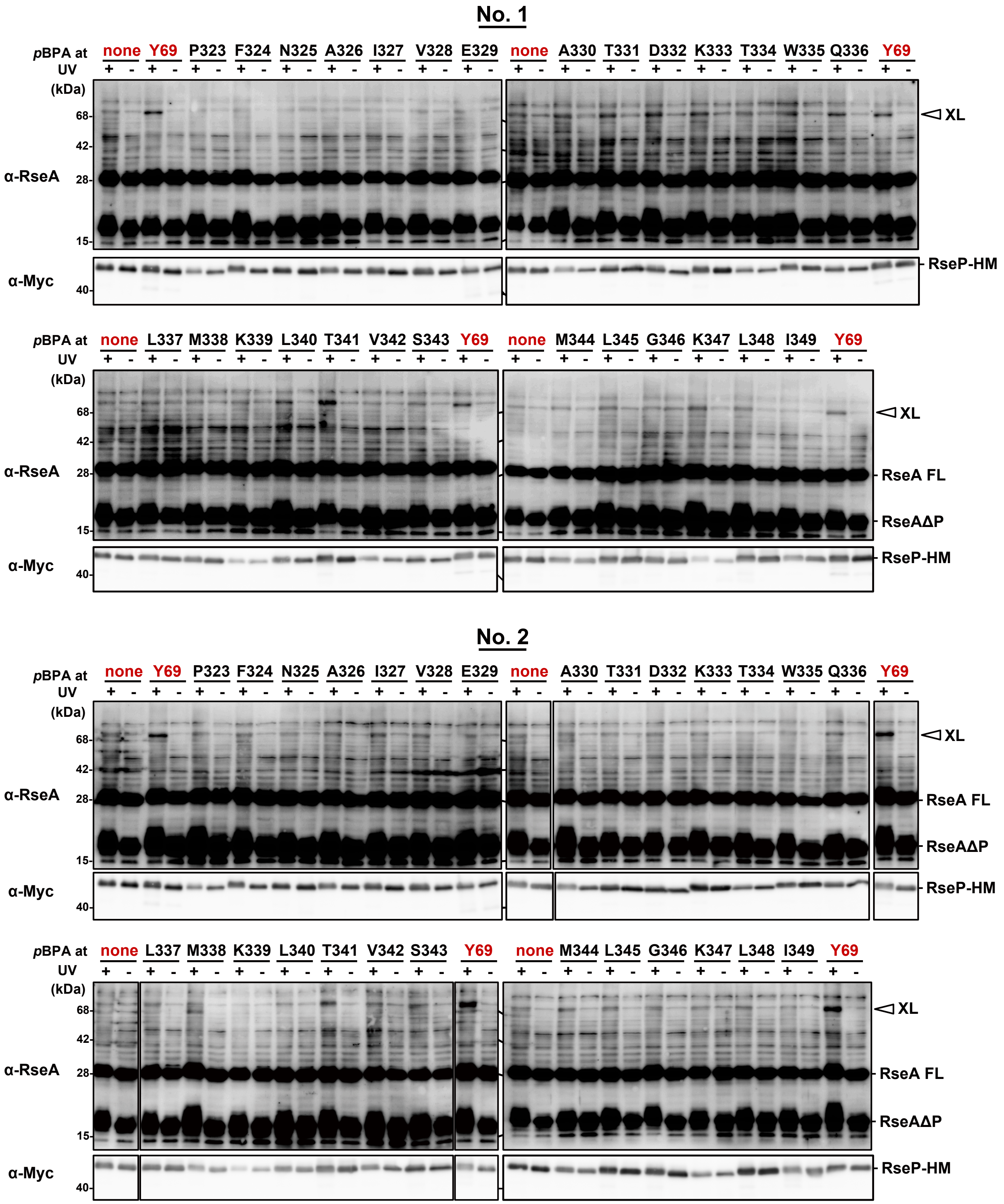


**Supplementary Figure S8. Systematic *in vivo* photo-crosslinking of the H1 segment.** *In vivo* photo-crosslinking between RseP H1 and RseA. KA418/pEVOL-pBpF were transformed with a plasmid encoding RseP(E23Q)-HM without (pKA52, none) or with an amber mutation at the indicated position. Cells were grown at 30°C in M9-based medium supplemented with 0.5 mM *p*BPA for 4 h and UV-irradiated for 10 min (UV+) or not (UV-). Whole cell lysates were analyzed as in Figure S7. RseP (Y69*p*BPA), which has a *p*BPA at the position of Tyr-69 in MRE β-loop, has been shown to be crosslinked with RseA and used as a positive control for crosslinking (Akiyama et al., 2015). None indicates RseP with no *p*BPA used as a negative control. Both controls were loaded in each gel for comparison of the band mobility and intensity (*labeled in red*). The results of the two independent experiments (No. 1 and No. 2) are shown.

## 2 Supplementary Methods

### Strains, plasmids and oligonucleotides

*Escherichia coli* K-12 strains, plasmids and oligonucleotides used in this work are listed in Supplementary Tables S1, S2 and S3, respectively. Construction of the individual strain and plasmids are described as follows. AD2473 was constructed by transferring the Δ*degS*::*tet* marker from a *degS*::*tet* derivative of AB1157/pKM201 (AD1835) (Kanehara et al., 2002) into AD2469 (Saito et al., 2011) by P1 transduction.

Plasmids encoding RseP-His$_6$-Myc with a point mutation were constructed by standard site-directed mutagenesis using appropriate combinations of mutagenic primers and a template plasmid. To exchange *rseP-his*$_6$*-myc* derivatives with a mutation among the compatible vector plasmids, *e.g.*, the pUC118, pTWV228, pSTD689 or pMW118, a 1.5 kb-SacI/HindIII fragment of the original plasmids was inserted into the same site of other vector plasmids. Similarly, to exchange *rseP-his*$_6$*-myc* with the E23Q mutation, a 1.3 kb-BssHII/HindIII fragment of the original plasmids was inserted into the same site of the plasmid with E23Q mutation. To construct pTM132 (RseP-His$_6$-Myc, Cys-less), a 1.4 kb-SacI/ClaI fragment of pSTD892 was first cloned into the same site of pKK11 (RseP-His$_6$-Myc) to obtain pTM101. Then, a 1.5 kb-SacI/HindIII fragment of pTM101 was recloned into the same site of pSTD689. A series of the RseP mono cysteine mutant plasmids, pTM102 ~ pTM128, were derivatives of pTM101, and pTM133 ~ pTM159 were derivatives of pTM132. The RseP double mutant plasmids, pTM517, pTM519, pTM521, pTM525, pTM527, pTM603 and pTM605, were obtained by a mutator-induced mutagenesis of pTM235 (RseP A326W) (see below). Their derivatives without the A326W mutation (pTM516, pTM518, pTM520, pTM524, pTM526, pTM602 and pTM604) were constructed by converting the codon for Trp-326 to the wild type codon by site directed mutagenesis of the above double mutant plasmids. pTM320 (RseP(ΔH1)-His$_6$-Myc) was constructed by site-directed mutagenesis of pKK11 to delete the Pro323-Ile349 region of RseP using primers P1/P2. The plasmids encoding the *E. coli* RseP H1 chimeras that possess other exogenous sequences in place of the H1 segment (Pro323-Ile349) region were constructed using In-Fusion HD Cloning Kit (Takara Bio Inc.) as follows. To construct pTM365 (RseP H1(Aae-H1)-His$_6$-Myc), the coding sequence for the H1 segment of *Aquifex aeolicus* RseP orthologues (Phe300-Ile326) were amplified from a plasmid carrying the *AarseP* gene (UniProtKB accession number: O67776) of *A. aeolicus* VF5 strain (Hizukuri

et al., 2014) using primers P3/P4 and ligated with the vector fragment amplified from pKK11 (*Ec*RseP-$His_6$-Myc) plasmid with primers P7/P8. Similarly, for construction of pTM438 (RseP H1(RseA-peri)-$His_6$-Myc), the sequence coding a hydrophilic helix segment in the periplasmic region of *Ec*RseA (Gln162-Leu186) was amplified from a plasmid pKK55 (*Ec*RseA) using primers P5/P6 and ligated with pKK11-derived vector fragment amplified with primers P7/P8. For construction of pTM427 (RseP H1(RNaseE-segA)-$His_6$-Myc), the sequence coding the segment A of *Ec*RNaseE (Pro568-Phe582) was amplified from a genome of strain JM109 using primers P17/P18 and ligated with a pKK11-derived vector fragment. To construct pTM366 (RseP H1(Bbr-H1)-$His_6$-Myc), the sequence coding the H1 segment (Val315-Val341) of *Bordetella bronchiseptica* RseP orthologues (UniProtKB accession number: A0A0H3LMM6) of *B. bronchiseptica* RB50 strain was chemically synthesized as two pair of complementally oligonucleotides (P9/P10 and P11/P12) and ligated with a pKK11-derived vector fragment. pTM367 (RseP H1(Vch-H1)-$His_6$-Myc) was constructed in the same way as pTM366; the coding sequence of the H1 segment (Val325-Leu351) of *Vibrio cholerae* RseP orthologues (UniProtKB accession number: Q9KPV9) of *V. cholerae* O1 El Tor N16961 strain was chemically synthesized as two pair of complementally oligonucleotides (P13/P14 and P15/P16) and ligated with a pKK11-derived vector fragment. To construct pTM685 (HA-RseA148), the codon for Ser-149 in *ha-rseA* of pKK55 was replaced with an ochre codon by standard site-directed mutagenesis using appropriate primers.

# 3 Supplementary Tables

## Table S1. *E. coli* strains.

| Strains | Relevant genotype | References or sources |
|---|---|---|
| AD16 | Δ*pro-lac thi* / F' *lacI*$^q$ *Z*ΔM15 *Y*$^+$ *pro*$^+$ | (Kihara et al., 1995) |
| AD1835 | AB1157, Δ*degS*::*tet* / pKM201 | (Kanehara et al., 2002) |
| KK211 | AD16, Δ*rseA*::*cat* Δ*rseP*::*kan* | (Kanehara et al., 2002) |
| AD1840 | AD16, Δ*rseA*::*cat* Δ*rseP*::*kan* Δ*degS*::*tet* | (Kanehara et al., 2002) |
| KA306 | AD16 Δ*rseA* Δ*rseP*::*kan* Δ*clpP*::*cat* | (Akiyama et al., 2015) |
| KK31 | AD16, Δ*rseP*::*kan* Δ(*srl-recA*)*306*::Tn*10* /pKK6 ($P_{BAD}$-*rseP*) | (Kanehara et al., 2001) |
| MC4100 | *araD139* Δ(*argF-lac*)*U169 rpsL150 relA1 flbB5301 deoC1 ptsF25 rbsR* | (Silhavy et al., 1984) |
| CU141 | MC4100 / F'*lacI*$^q$ *lacZYA*$^+$ | (Akiyama et al., 1994) |
| KK374 | CU141, Δ*rseA*::*cat* Δ*rseP*::*kan* Δ*degS*::*tet* | (Akiyama et al., 2004) |
| KA418 | CU141, Δ*ompA* Δ*ompC* Δ*rseP*::*kan* | (Akiyama et al., 2015) |
| KA438 | CU141, Δ*ompA* Δ*ompC* Δ*rseP*::*kan* Δ*degS*::*tet* | (Akiyama et al., 2015) |
| MC1061 | *araD* Δ(*ara-leu*)*7697* Δ(*codB-lacI*) *galK16 galE15 mcrA0 relA1 rpsL150 spoT1 mcrB9999 hsdR2* | (Casadaban and Cohen, 1980) |
| CAG16037 | MC1061, Φλ[*rpoHP3-lacZ*] | (Mecsas et al., 1993) |
| AD2469 | CAG16037, Δ*ompA* Δ*ompC* Δ*rseP*::*kan* | (Saito et al., 2011) |
| AD2473 | AD2469, Δ*degS*::*tet* | this study |
| TR71 | MC4100, λRS45[*rpoHP3-lacZ*] | A gift of T. J. Silhavy. |
| XL1-Red | *endA1 gyrA96 thi-1 hsdR17 supE44 relA1 lac mutD5 mutS mutT Tn10* (Tet$^r$) | Agilent |
| KD1087 | *Δ(tonB-trpA,B) leu argE his spcA mutD5* | (Degnen and Cox, 1974) |

## Table S2. Plasmids.

| Plasmids | Vector | Encoded proteins or descriptions[a] | References or sources |
|---|---|---|---|
| pBAD33 | | pACYC184-based vector; $P_{BAD}$, $Cm^R$ | (Guzman et al., 1995) |
| pMW118 | | pSC101-based vector; $P_{lac}$, $Amp^R$ | Nippon Gene |
| pSTD689 | | pACYC184-based vector; $P_{lac}$, $Spc^R$ | (Kanehara et al., 2003) |
| pSTV29 | | pACYC184-based vector; $P_{lac}$, $Cm^R$ | Takara Bio |
| pTWV228 | | pBR322-based vector; $P_{lac}$, $Amp^R$ | Takara Bio |
| pTYE007 | | pBlueScript SK(-)-based vector; carring *his*$_6$-*myc* tag, $P_{lac}$, $Amp^R$ | (Akiyama et al., 1995) |
| pUC118 | | pBR322-based vector; $P_{lac}$, $Amp^R$ | Takara Bio |
| pEVOL-pBpF | | p15A-derivative encoding mutant *M. jannaschii* aminoacyl-tRNA synthetase and suppressor tRNA for pBPA incorporation; $Cm^R$ | (Young et al., 2010) |
| pKA52 | pUC118 | RseP-His$_6$-Myc, E23Q | (Akiyama et al., 2015) |
| pKA81 | pUC118 | RseP-His$_6$-Myc, E23Q, Y69amber | (Akiyama et al., 2015) |
| pKA107 | pUC118 | RseP-His$_6$-Myc, Y69amber | (Akiyama et al., 2015) |
| pKK6 | pBAD33 | RseP | (Kanehara et al., 2001) |
| pKK10 | pMW118 | RseP-His$_6$-Myc | (Kanehara et al., 2001) |
| pKK11 | pTWV228 | RseP-His$_6$-Myc | (Kanehara et al., 2001) |
| pKK49 | pUC118 | RseP-His$_6$-Myc | (Akiyama et al., 2004) |
| pKK55 | pTWV228 | HA-RseA | (Kanehara et al., 2002) |
| pSTD343 | pSTV29 | LacI | (Sakoh et al., 2005) |
| pSTD691 | pSTD689 | HA-RseA | (Kanehara et al., 2002) |

| | | | |
|---|---|---|---|
| pSTD892 | pTYE007 | RseP-His$_6$-Myc, C33A, C427A (= Cys-less) | (Koide et al., 2007) |
| pTM11 | pTWV228 | RseP-His$_6$-Myc, A326K | this study |
| pTM101 | pTWV228 | RseP-His$_6$-Myc, Cys-less | this study |
| pTM102 | pTWV228 | RseP-His$_6$-Myc, Cys-less, P323C | this study |
| pTM103 | pTWV228 | RseP-His$_6$-Myc, Cys-less, F324C | this study |
| pTM104 | pTWV228 | RseP-His$_6$-Myc, Cys-less, N325C | this study |
| pTM105 | pTWV228 | RseP-His$_6$-Myc, Cys-less, A326C | this study |
| pTM106 | pTWV228 | RseP-His$_6$-Myc, Cys-less, I327C | this study |
| pTM107 | pTWV228 | RseP-His$_6$-Myc, Cys-less, V328C | this study |
| pTM108 | pTWV228 | RseP-His$_6$-Myc, Cys-less, E329C | this study |
| pTM109 | pTWV228 | RseP-His$_6$-Myc, Cys-less, A330C | this study |
| pTM110 | pTWV228 | RseP-His$_6$-Myc, Cys-less, T331C | this study |
| pTM111 | pTWV228 | RseP-His$_6$-Myc, Cys-less, D332C | this study |
| pTM112 | pTWV228 | RseP-His$_6$-Myc, Cys-less, K333C | this study |
| pTM113 | pTWV228 | RseP-His$_6$-Myc, Cys-less, T334C | this study |
| pTM114 | pTWV228 | RseP-His$_6$-Myc, Cys-less, W335C | this study |
| pTM115 | pTWV228 | RseP-His$_6$-Myc, Cys-less, Q336C | this study |
| pTM116 | pTWV228 | RseP-His$_6$-Myc, Cys-less, L337C | this study |
| pTM117 | pTWV228 | RseP-His$_6$-Myc, Cys-less, M338C | this study |
| pTM118 | pTWV228 | RseP-His$_6$-Myc, Cys-less, K339C | this study |
| pTM119 | pTWV228 | RseP-His$_6$-Myc, Cys-less, L340C | this study |
| pTM120 | pTWV228 | RseP-His$_6$-Myc, Cys-less, T341C | this study |
| pTM121 | pTWV228 | RseP-His$_6$-Myc, Cys-less, V342C | this study |
| pTM122 | pTWV228 | RseP-His$_6$-Myc, Cys-less, S343C | this study |
| pTM123 | pTWV228 | RseP-His$_6$-Myc, Cys-less, M344C | this study |
| pTM124 | pTWV228 | RseP-His$_6$-Myc, Cys-less, L345C | this study |
| pTM125 | pTWV228 | RseP-His$_6$-Myc, Cys-less, G346C | this study |
| pTM126 | pTWV228 | RseP-His$_6$-Myc, Cys-less, K347C | this study |
| pTM127 | pTWV228 | RseP-His$_6$-Myc, Cys-less, L348C | this study |
| pTM128 | pTWV228 | RseP-His$_6$-Myc, Cys-less, I349C | this study |
| pTM132 | pSTD689 | RseP-His$_6$-Myc, Cys-less | this study |
| pTM133 | pSTD689 | RseP-His$_6$-Myc, Cys-less, P323C | this study |
| pTM134 | pSTD689 | RseP-His$_6$-Myc, Cys-less, F324C | this study |
| pTM135 | pSTD689 | RseP-His$_6$-Myc, Cys-less, N325C | this study |
| pTM136 | pSTD689 | RseP-His$_6$-Myc, Cys-less, A326C | this study |
| pTM137 | pSTD689 | RseP-His$_6$-Myc, Cys-less, I327C | this study |
| pTM138 | pSTD689 | RseP-His$_6$-Myc, Cys-less, V328C | this study |
| pTM139 | pSTD689 | RseP-His$_6$-Myc, Cys-less, E329C | this study |
| pTM140 | pSTD689 | RseP-His$_6$-Myc, Cys-less, A330C | this study |
| pTM141 | pSTD689 | RseP-His$_6$-Myc, Cys-less, T331C | this study |
| pTM142 | pSTD689 | RseP-His$_6$-Myc, Cys-less, D332C | this study |
| pTM143 | pSTD689 | RseP-His$_6$-Myc, Cys-less, K333C | this study |
| pTM144 | pSTD689 | RseP-His$_6$-Myc, Cys-less, T334C | this study |
| pTM145 | pSTD689 | RseP-His$_6$-Myc, Cys-less, W335C | this study |
| pTM146 | pSTD689 | RseP-His$_6$-Myc, Cys-less, Q336C | this study |
| pTM147 | pSTD689 | RseP-His$_6$-Myc, Cys-less, L337C | this study |
| pTM148 | pSTD689 | RseP-His$_6$-Myc, Cys-less, M338C | this study |
| pTM149 | pSTD689 | RseP-His$_6$-Myc, Cys-less, K339C | this study |
| pTM150 | pSTD689 | RseP-His$_6$-Myc, Cys-less, L340C | this study |
| pTM151 | pSTD689 | RseP-His$_6$-Myc, Cys-less, T341C | this study |
| pTM152 | pSTD689 | RseP-His$_6$-Myc, Cys-less, V342C | this study |
| pTM153 | pSTD689 | RseP-His$_6$-Myc, Cys-less, S343C | this study |
| pTM154 | pSTD689 | RseP-His$_6$-Myc, Cys-less, M344C | this study |
| pTM155 | pSTD689 | RseP-His$_6$-Myc, Cys-less, L345C | this study |
| pTM156 | pSTD689 | RseP-His$_6$-Myc, Cys-less, G346C | this study |
| pTM157 | pSTD689 | RseP-His$_6$-Myc, Cys-less, K347C | this study |
| pTM158 | pSTD689 | RseP-His$_6$-Myc, Cys-less, L348C | this study |

| | | | |
|---|---|---|---|
| pTM159 | pSTD689 | RseP-His$_6$-Myc, Cys-less, I349C | this study |
| pTM231 | pTWV228 | RseP-His$_6$-Myc, A326C | this study |
| pTM232 | pTWV228 | RseP-His$_6$-Myc, A326E | this study |
| pTM233 | pTWV228 | RseP-His$_6$-Myc, A326G | this study |
| pTM234 | pTWV228 | RseP-His$_6$-Myc, A326L | this study |
| pTM235 | pTWV228 | RseP-His$_6$-Myc, A326W | this study |
| pTM320 | pTWV228 | RseP-His$_6$-Myc, ΔH1 | this study |
| pTM324 | pSTD689 | RseP-His$_6$-Myc, ΔH1 | this study |
| pTM341 | pUC118 | RseP-His$_6$-Myc, P323amber | this study |
| pTM342 | pUC118 | RseP-His$_6$-Myc, A326amber | this study |
| pTM343 | pUC118 | RseP-His$_6$-Myc, D332amber | this study |
| pTM344 | pUC118 | RseP-His$_6$-Myc, W335amber | this study |
| pTM345 | pUC118 | RseP-His$_6$-Myc, T341amber | this study |
| pTM346 | pUC118 | RseP-His$_6$-Myc, L345amber | this study |
| pTM347 | pUC118 | RseP-His$_6$-Myc, K347amber | this study |
| pTM365 | pTWV228 | RseP-His$_6$-Myc, H1(Aae-H1) | this study |
| pTM366 | pTWV228 | RseP-His$_6$-Myc, H1(Bbr-H1) | this study |
| pTM367 | pTWV228 | RseP-His$_6$-Myc, H1(Vch-H1) | this study |
| pTM368 | pSTD689 | RseP-His$_6$-Myc, H1(Aae-H1) | this study |
| pTM369 | pSTD689 | RseP-His$_6$-Myc, H1(Bbr-H1) | this study |
| pTM370 | pSTD689 | RseP-His$_6$-Myc, H1(Vch-H1) | this study |
| pTM371 | pUC118 | RseP-His$_6$-Myc, E23Q, P323amber | this study |
| pTM372 | pUC118 | RseP-His$_6$-Myc, E23Q, A326amber | this study |
| pTM373 | pUC118 | RseP-His$_6$-Myc, E23Q, D332amber | this study |
| pTM374 | pUC118 | RseP-His$_6$-Myc, E23Q, W335amber | this study |
| pTM375 | pUC118 | RseP-His$_6$-Myc, E23Q, T341amber | this study |
| pTM376 | pUC118 | RseP-His$_6$-Myc, E23Q, L345amber | this study |
| pTM377 | pUC118 | RseP-His$_6$-Myc, E23Q, K347amber | this study |
| pTM393 | pTWV228 | RseP-His$_6$-Myc, M338W | this study |
| pTM398 | pTWV228 | RseP-His$_6$-Myc, Q336P | this study |
| pTM410 | pSTD689 | RseP-His$_6$-Myc, M338W | this study |
| pTM415 | pSTD689 | RseP-His$_6$-Myc, Q336P | this study |
| pTM424 | pTWV228 | RseP-His$_6$-Myc, H1(RseA-peri) | this study |
| pTM427 | pTWV228 | RseP-His$_6$-Myc, H1(RNaseE-segA) | this study |
| pTM438 | pSTD689 | RseP-His$_6$-Myc, H1(RseA-peri) | this study |
| pTM441 | pSTD689 | RseP-His$_6$-Myc, H1(RNaseE-segA) | this study |
| pTM470 | pUC118 | RseP-His$_6$-Myc, ΔH1 | this study |
| pTM477 | pUC118 | RseP-His$_6$-Myc, E23Q, ΔH1 | this study |
| pTM500 | pTWV228 | RseP-His$_6$-Myc, A326P | this study |
| pTM501 | pTWV228 | RseP-His$_6$-Myc, E329P | this study |
| pTM502 | pTWV228 | RseP-His$_6$-Myc, D332P | this study |
| pTM503 | pTWV228 | RseP-His$_6$-Myc, W335P | this study |
| pTM504 | pTWV228 | RseP-His$_6$-Myc, M338P | this study |
| pTM505 | pTWV228 | RseP-His$_6$-Myc, T341P | this study |
| pTM506 | pTWV228 | RseP-His$_6$-Myc, M344P | this study |
| pTM507 | pTWV228 | RseP-His$_6$-Myc, K347P | this study |
| pTM508 | pSTD689 | RseP-His$_6$-Myc, A326P | this study |
| pTM509 | pSTD689 | RseP-His$_6$-Myc, E329P | this study |
| pTM510 | pSTD689 | RseP-His$_6$-Myc, D332P | this study |
| pTM511 | pSTD689 | RseP-His$_6$-Myc, W335P | this study |
| pTM512 | pSTD689 | RseP-His$_6$-Myc, M338P | this study |
| pTM513 | pSTD689 | RseP-His$_6$-Myc, T341P | this study |
| pTM514 | pSTD689 | RseP-His$_6$-Myc, M344P | this study |
| pTM515 | pSTD689 | RseP-His$_6$-Myc, K347P | this study |
| pTM516 | pTWV228 | RseP-His$_6$-Myc, P306S | this study |
| pTM517 | pTWV228 | RseP-His$_6$-Myc, P306S, A326W | this study |
| pTM518 | pTWV228 | RseP-His$_6$-Myc, S138A | this study |

| | | | |
|---|---|---|---|
| pTM519 | pTWV228 | RseP-$His_6$-Myc, S138A, A326W | this study |
| pTM520 | pTWV228 | RseP-$His_6$-Myc, I215V | this study |
| pTM521 | pTWV228 | RseP-$His_6$-Myc, I215V, A326W | this study |
| pTM524 | pTWV228 | RseP-$His_6$-Myc, P147L | this study |
| pTM525 | pTWV228 | RseP-$His_6$-Myc, P147L, A326W | this study |
| pTM526 | pTWV228 | RseP-$His_6$-Myc, W257R | this study |
| pTM527 | pTWV228 | RseP-$His_6$-Myc, W257R, A326W | this study |
| pTM540 | pTWV228 | RseP-$His_6$-Myc, L151P, A326W | this study |
| pTM566 | pTWV228 | RseP-$His_6$-Myc, F324P | this study |
| pTM567 | pTWV228 | RseP-$His_6$-Myc, N325P | this study |
| pTM568 | pTWV228 | RseP-$His_6$-Myc, I327P | this study |
| pTM569 | pTWV228 | RseP-$His_6$-Myc, V328P | this study |
| pTM570 | pTWV228 | RseP-$His_6$-Myc, A330P | this study |
| pTM571 | pTWV228 | RseP-$His_6$-Myc, T331P | this study |
| pTM572 | pTWV228 | RseP-$His_6$-Myc, K333P | this study |
| pTM573 | pTWV228 | RseP-$His_6$-Myc, T334P | this study |
| pTM574 | pTWV228 | RseP-$His_6$-Myc, L337P | this study |
| pTM575 | pTWV228 | RseP-$His_6$-Myc, K339P | this study |
| pTM576 | pTWV228 | RseP-$His_6$-Myc, L340P | this study |
| pTM577 | pTWV228 | RseP-$His_6$-Myc, V342P | this study |
| pTM578 | pTWV228 | RseP-$His_6$-Myc, S343P | this study |
| pTM579 | pTWV228 | RseP-$His_6$-Myc, L345P | this study |
| pTM580 | pTWV228 | RseP-$His_6$-Myc, G346P | this study |
| pTM581 | pTWV228 | RseP-$His_6$-Myc, L348P | this study |
| pTM582 | pTWV228 | RseP-$His_6$-Myc, I349P | this study |
| pTM583 | pSTD689 | RseP-$His_6$-Myc, F324P | this study |
| pTM584 | pSTD689 | RseP-$His_6$-Myc, N325P | this study |
| pTM585 | pSTD689 | RseP-$His_6$-Myc, I327P | this study |
| pTM586 | pSTD689 | RseP-$His_6$-Myc, V328P | this study |
| pTM587 | pSTD689 | RseP-$His_6$-Myc, A330P | this study |
| pTM588 | pSTD689 | RseP-$His_6$-Myc, T331P | this study |
| pTM589 | pSTD689 | RseP-$His_6$-Myc, K333P | this study |
| pTM590 | pSTD689 | RseP-$His_6$-Myc, T334P | this study |
| pTM591 | pSTD689 | RseP-$His_6$-Myc, L337P | this study |
| pTM592 | pSTD689 | RseP-$His_6$-Myc, K339P | this study |
| pTM593 | pSTD689 | RseP-$His_6$-Myc, L340P | this study |
| pTM594 | pSTD689 | RseP-$His_6$-Myc, V342P | this study |
| pTM595 | pSTD689 | RseP-$His_6$-Myc, S343P | this study |
| pTM596 | pSTD689 | RseP-$His_6$-Myc, L345P | this study |
| pTM597 | pSTD689 | RseP-$His_6$-Myc, G346P | this study |
| pTM598 | pSTD689 | RseP-$His_6$-Myc, L348P | this study |
| pTM599 | pSTD689 | RseP-$His_6$-Myc, I349P | this study |
| pTM602 | pTWV228 | RseP-$His_6$-Myc, K316E | this study |
| pTM603 | pTWV228 | RseP-$His_6$-Myc, K316E, A326W | this study |
| pTM604 | pTWV228 | RseP-$His_6$-Myc, K333E | this study |
| pTM605 | pTWV228 | RseP-$His_6$-Myc, K333E, A326W | this study |
| pTM615 | pUC118 | RseP-$His_6$-Myc, Q336amber | this study |
| pTM616 | pUC118 | RseP-$His_6$-Myc, L337amber | this study |
| pTM617 | pUC118 | RseP-$His_6$-Myc, M338amber | this study |
| pTM618 | pUC118 | RseP-$His_6$-Myc, K339amber | this study |
| pTM619 | pUC118 | RseP-$His_6$-Myc, L340amber | this study |
| pTM620 | pUC118 | RseP-$His_6$-Myc, V342amber | this study |
| pTM621 | pUC118 | RseP-$His_6$-Myc, S343amber | this study |
| pTM622 | pUC118 | RseP-$His_6$-Myc, M344amber | this study |
| pTM623 | pUC118 | RseP-$His_6$-Myc, E23Q, Q336amber | this study |
| pTM624 | pUC118 | RseP-$His_6$-Myc, E23Q, L337amber | this study |
| pTM625 | pUC118 | RseP-$His_6$-Myc, E23Q, M338amber | this study |

| | | | |
|---|---|---|---|
| pTM626 | pUC118 | RseP-$His_6$-Myc, E23Q, K339amber | this study |
| pTM627 | pUC118 | RseP-$His_6$-Myc, E23Q, L340amber | this study |
| pTM628 | pUC118 | RseP-$His_6$-Myc, E23Q, V342amber | this study |
| pTM629 | pUC118 | RseP-$His_6$-Myc, E23Q, S343amber | this study |
| pTM630 | pUC118 | RseP-$His_6$-Myc, E23Q, M344amber | this study |
| pTM635 | pUC118 | RseP-$His_6$-Myc, F324amber | this study |
| pTM636 | pUC118 | RseP-$His_6$-Myc, N325amber | this study |
| pTM637 | pUC118 | RseP-$His_6$-Myc, I327amber | this study |
| pTM638 | pUC118 | RseP-$His_6$-Myc, V328amber | this study |
| pTM639 | pUC118 | RseP-$His_6$-Myc, E329amber | this study |
| pTM640 | pUC118 | RseP-$His_6$-Myc, A330amber | this study |
| pTM641 | pUC118 | RseP-$His_6$-Myc, T331amber | this study |
| pTM642 | pUC118 | RseP-$His_6$-Myc, K333amber | this study |
| pTM643 | pUC118 | RseP-$His_6$-Myc, T334amber | this study |
| pTM644 | pUC118 | RseP-$His_6$-Myc, G346amber | this study |
| pTM645 | pUC118 | RseP-$His_6$-Myc, L348amber | this study |
| pTM646 | pUC118 | RseP-$His_6$-Myc, I349amber | this study |
| pTM647 | pUC118 | RseP-$His_6$-Myc, E23Q, F324amber | this study |
| pTM648 | pUC118 | RseP-$His_6$-Myc, E23Q, N325amber | this study |
| pTM649 | pUC118 | RseP-$His_6$-Myc, E23Q, I327amber | this study |
| pTM650 | pUC118 | RseP-$His_6$-Myc, E23Q, V328amber | this study |
| pTM651 | pUC118 | RseP-$His_6$-Myc, E23Q, E329amber | this study |
| pTM652 | pUC118 | RseP-$His_6$-Myc, E23Q, A330amber | this study |
| pTM653 | pUC118 | RseP-$His_6$-Myc, E23Q, T331amber | this study |
| pTM654 | pUC118 | RseP-$His_6$-Myc, E23Q, K333amber | this study |
| pTM655 | pUC118 | RseP-$His_6$-Myc, E23Q, T334amber | this study |
| pTM656 | pUC118 | RseP-$His_6$-Myc, E23Q, G346amber | this study |
| pTM657 | pUC118 | RseP-$His_6$-Myc, E23Q, L348amber | this study |
| pTM658 | pUC118 | RseP-$His_6$-Myc, E23Q, I349amber | this study |
| pTM659 | pTWV228 | RseP-$His_6$-Myc, M338A | this study |
| pTM660 | pTWV228 | RseP-$His_6$-Myc, M338C | this study |
| pTM661 | pTWV228 | RseP-$His_6$-Myc, M338E | this study |
| pTM662 | pTWV228 | RseP-$His_6$-Myc, M338G | this study |
| pTM663 | pTWV228 | RseP-$His_6$-Myc, M338K | this study |
| pTM665 | pSTD689 | RseP-$His_6$-Myc, M338A | this study |
| pTM666 | pSTD689 | RseP-$His_6$-Myc, M338C | this study |
| pTM667 | pSTD689 | RseP-$His_6$-Myc, M338E | this study |
| pTM668 | pSTD689 | RseP-$His_6$-Myc, M338G | this study |
| pTM669 | pSTD689 | RseP-$His_6$-Myc, M338K | this study |
| pTM685 | pSTD689 | HA-RseA148 | this study |
| pYGF11 | pTWV228 | RseP-$His_6$-Myc, A326V | (Inaba et al., 2008) |
| pYGF13 | pTWV228 | RseP-$His_6$-Myc, L151P | (Inaba et al., 2008) |
| pYH9 | pSTD689 | RseP-$His_6$-Myc | (Hizukuri and Akiyama, 2012) |
| pYH19 | pTWV228 | HA-MBP-RseA148 | (Hizukuri and Akiyama, 2012) |
| pYH20 | pTWV228 | HA-MBP-RseA(LY1)148 | (Hizukuri and Akiyama, 2012) |
| pYH124 | pSTD689 | HA-MBP-RseA(LY1)148 | (Hizukuri and Akiyama, 2012) |

[a] $Amp^R$, ampicillin resistant; $Spc^R$, spectinomycin resistant; $Km^R$, kanamycin resistant; $Cm^R$, chloramphenicol resistant.

## Table S3. Primers and oligonucleotides

| Serial No. | Primer name | Sequence |
|---|---|---|
| P1 | EcRseP d323-349 (+) | GTATAAAGTTGTACGCCAGTATGGGACCGGTGATGTGAAACTG AACAACC |
| P2 | EcRseP d323-349 (-) | GGTTGTTCAGTTTCACATCACCGGTCCCATACTGGCGTACAACT TTATAC |
| P3 | Aae-H1 connection primer (+) | GTACGCCAGTATGGGTTCGGAGAGGCTCTG |
| P4 | Aae-H1 connection primer (-) | TTTCACATCACCGGTTATAAGCCCTGCTAT |
| P5 | RseA-peri helix connection (+) | GTACGCCAGTATGGGCAACAGCAGCAGGTA |
| P6 | RseA-peri helix connection (-) | TTTCACATCACCGGTGAGTCGGCGTTGCAG |
| P7 | RseP H1vector for | ACCGGTGATGTGAAACTGAACAACC |
| P8 | RseP H1vector rev | CCCATACTGGCGTACAACTTTATAC |
| P9 | Vch-H1_1 (+) | GTACGCCAGTATGGGGTTTTCGAATCTCTGGGTAAAGCTGTTG AAAAATCTGGTCAGGTTATCG |
| P10 | Vch-H1_1 (-) | CGATAACCTGACCAGATTTTTCAACAGCTTTACCCAGAGATTCG AAAACCCCATACTGGCGTAC |
| P11 | Vch-H1_2 (+) | CTGGTCAGGTTATCGACCTGACCGTTTCTATGCTGAAAAAACT GCTGACCGGTGATGTGAAA |
| P12 | Vch-H1_2 (-) | TTTCACATCACCGGTCAGCAGTTTTTTCAGCATAGAAACGGTC AGGTCGATAACCTGACCAG |
| P13 | Bbr-H1_1 (+) | GTACGCCAGTATGGGGTTATCGACTCTGTTTGGCGTGGTGCTCA GCGTACCTGGGACACCGCTTG |
| P14 | Bbr-H1_1 (-) | CAAGCGGTGTCCCAGGTACGCTGAGCACCACGCCAAACAGAG TCGATAACCCCATACTGGCGTAC |
| P15 | Bbr-H1_2 (+) | CTGGGACACCGCTTGGCTGTCTCTGCGTATGATGGGTCGTATGG TTACCGGTGATGTGAAA |
| P16 | Bbr-H1_2 (-) | TTTCACATCACCGGTAACCATACGACCCATCATACGCAGAGAC AGCCAAGCGGTGTCCCAG |
| P17 | rne-segA connection (+) | GTACGCCAGTATGGGCCTGGGCTGTTGAGC |
| P18 | rne-segA connection (-) | TTTCACATCACCGGTGAACAGCGCTTTCAG |

## 4 References


Akiyama, K., Mizuno, S., Hizukuri, Y., Mori, H., Nogi, T., and Akiyama, Y. (2015). Roles of the membrane-reentrant β-hairpin-like loop of RseP protease in selective substrate cleavage. *eLife* 4, e08928. doi: 10.7554/eLife.08928

Akiyama, Y., Kanehara, K., and Ito, K. (2004). RseP (YaeL), an *Escherichia coli* RIP protease, cleaves transmembrane sequences. *EMBO J.* 23, 4434-4442. doi: 10.1038/sj.emboj.7600449

Akiyama, Y., Ogura, T., and Ito, K. (1994). Involvement of FtsH in protein assembly into and through the membrane. I. Mutations that reduce retention efficiency of a cytoplasmic reporter. *J. Biol. Chem.* 269, 5218-5224.

Akiyama, Y., Yoshihisa, T., and Ito, K. (1995). FtsH, a membrane-bound ATPase, forms a complex in the cytoplasmic membrane of *Escherichia coli*. *J. Biol. Chem.* 270, 23485-23490. doi: 10.1074/jbc.270.40.23485

Casadaban, M.J., and Cohen, S.N. (1980). Analysis of gene control signals by DNA fusion and cloning in *Escherichia coli*. *J. Mol. Biol.* 138, 179-207. doi: 10.1016/0022-2836(80)90283-1

Degnen, G.E., and Cox, E.C. (1974). Conditional mutator gene in Escherichia coli: isolation, mapping, and effector studies. *J. Bacteriol.* 117, 477-487. doi: 10.1128/JB.117.2.477-487.1974

El-Gebali, S., Mistry, J., Bateman, A., Eddy, S.R., Luciani, A., Potter, S.C., et al. (2019). The Pfam protein families database in 2019. *Nucleic Acids Res.* 47, D427-D432. doi: 10.1093/nar/gky995

Guzman, L.M., Belin, D., Carson, M.J., and Beckwith, J. (1995). Tight regulation, modulation, and high-level expression by vectors containing the arabinose $P_{BAD}$ promoter. *J. Bacteriol.* 177, 4121-4130. doi: 10.1128/jb.177.14.4121-4130.1995

Hizukuri, Y., and Akiyama, Y. (2012). PDZ domains of RseP are not essential for sequential cleavage of RseA or stress-induced $\sigma^E$ activation *in vivo*. *Mol. Microbiol.* 86, 1232-1245. doi: 10.1111/mmi.12053

Hizukuri, Y., Oda, T., Tabata, S., Tamura-Kawakami, K., Oi, R., Sato, M., et al. (2014). A structure-based model of substrate discrimination by a noncanonical PDZ tandem in the intramembrane-cleaving protease RseP. *Structure* 22, 326-336. doi: 10.1016/j.str.2013.12.003

Inaba, K., Suzuki, M., Maegawa, K., Akiyama, S., Ito, K., and Akiyama, Y. (2008). A pair of circularly permutated PDZ domains control RseP, the S2P family intramembrane protease of *Escherichia coli*. *J. Biol. Chem.* 283, 35042-35052. doi: 10.1074/jbc.M806603200

Kanehara, K., Akiyama, Y., and Ito, K. (2001). Characterization of the *yaeL* gene product and its S2P-protease motifs in *Escherichia coli*. *Gene* 281, 71-79. doi: 10.1016/s0378-1119(01)00823-x

Kanehara, K., Ito, K., and Akiyama, Y. (2002). YaeL (EcfE) activates the $\sigma^E$ pathway of stress response through a site-2 cleavage of anti-$\sigma^E$, RseA. *Genes Dev.* 16, 2147-2155. doi: 10.1101/gad.1002302

Kanehara, K., Ito, K., and Akiyama, Y. (2003). YaeL proteolysis of RseA is controlled by the PDZ domain of YaeL and a Gln-rich region of RseA. *EMBO J.* 22, 6389-6398. doi: 10.1093/emboj/cdg602

Kihara, A., Akiyama, Y., and Ito, K. (1995). FtsH is required for proteolytic elimination of uncomplexed forms of SecY, an essential protein translocase subunit. *Proc. Natl. Acad. Sci. U. S. A.* 92, 4532-4536. doi: 10.1073/pnas.92.10.4532

Koide, K., Maegawa, S., Ito, K., and Akiyama, Y. (2007). Environment of the active site region of RseP, an *Escherichia coli* regulated intramembrane proteolysis protease, assessed by site-directed cysteine alkylation. *J. Biol. Chem.* 282, 4553-4560. doi: 10.1074/jbc.M607339200

Mecsas, J., Rouviere, P.E., Erickson, J.W., Donohue, T.J., and Gross, C.A. (1993). The activity of $\sigma^E$, an *Escherichia coli* heat-inducible σ-factor, is modulated by expression of outer-membrane

proteins. *Genes Dev.* 7, 2618-2628. doi: 10.1101/gad.7.12b.2618

Saito, A., Hizukuri, Y., Matsuo, E., Chiba, S., Mori, H., Nishimura, O., et al. (2011). Post-liberation cleavage of signal peptides is catalyzed by the site-2 protease (S2P) in bacteria. *Proc. Natl. Acad. Sci. U. S. A.* 108, 13740-13745. doi: 10.1073/pnas.1108376108

Sakoh, M., Ito, K., and Akiyama, Y. (2005). Proteolytic activity of HtpX, a membrane-bound and stress-controlled protease from *Escherichia coli*. *J. Biol. Chem.* 280, 33305-33310. doi: 10.1074/jbc.M506180200

Silhavy, T.J., Berman, M.L., and Enquist, L.W. (1984). Experiments with Gene Fusions. *Cold Spring Harbor, NewYork: Cold Spring Harbor Laboratory Press*.

Young, T.S., Ahmad, I., Yin, J.A., and Schultz, P.G. (2010). An enhanced system for unnatural amino acid mutagenesis in *E. coli*. *J. Mol. Biol.* 395, 361-374. doi: 10.1016/j.jmb.2009.10.030
